# PpERF17 alleviates peach fruit postharvest chilling injury under elevated CO_2_ by activating jasmonic acid and γ-aminobutyric acid biosynthesis

**DOI:** 10.1093/hr/uhaf014

**Published:** 2025-01-15

**Authors:** Shaojie Ai, Ling Liang, Mengfei Liu, Don Grierson, Kunsong Chen, Changjie Xu

**Affiliations:** College of Agriculture & Biotechnology, Zhejiang University, Zijingang Campus, 866 Yuhangtang Road, West Lake District, Hangzhou 310058, China; College of Agriculture & Biotechnology, Zhejiang University, Zijingang Campus, 866 Yuhangtang Road, West Lake District, Hangzhou 310058, China; Zhejiang Key Laboratory of Horticultural Crop Quality Improvement, Zhejiang University, Zijingang Campus, 866 Yuhangtang Road, West Lake District, Hangzhou 310058, China; College of Agriculture & Biotechnology, Zhejiang University, Zijingang Campus, 866 Yuhangtang Road, West Lake District, Hangzhou 310058, China; The State Agriculture Ministry Laboratory of Horticultural Plant Crop Growth and Development, Zhejiang University, Zijingang Campus, 866 Yuhangtang Road, West Lake District, Hangzhou 310058, China; College of Agriculture & Biotechnology, Zhejiang University, Zijingang Campus, 866 Yuhangtang Road, West Lake District, Hangzhou 310058, China; Plant Sciences Division, School of Biosciences, University of Nottingham, Sutton Bonington Campus, Loughborough, Leicestershire LE12 5RD, UK; College of Agriculture & Biotechnology, Zhejiang University, Zijingang Campus, 866 Yuhangtang Road, West Lake District, Hangzhou 310058, China; Zhejiang Key Laboratory of Horticultural Crop Quality Improvement, Zhejiang University, Zijingang Campus, 866 Yuhangtang Road, West Lake District, Hangzhou 310058, China; The State Agriculture Ministry Laboratory of Horticultural Plant Crop Growth and Development, Zhejiang University, Zijingang Campus, 866 Yuhangtang Road, West Lake District, Hangzhou 310058, China; College of Agriculture & Biotechnology, Zhejiang University, Zijingang Campus, 866 Yuhangtang Road, West Lake District, Hangzhou 310058, China; Zhejiang Key Laboratory of Horticultural Crop Quality Improvement, Zhejiang University, Zijingang Campus, 866 Yuhangtang Road, West Lake District, Hangzhou 310058, China; The State Agriculture Ministry Laboratory of Horticultural Plant Crop Growth and Development, Zhejiang University, Zijingang Campus, 866 Yuhangtang Road, West Lake District, Hangzhou 310058, China

## Abstract

Internal browning (IB) is a common chilling injury (CI) feature in peach fruit after prolonged cold storage. Our previous study demonstrated that low O_2_ and elevated CO_2_ (eCO_2_) condition of modified atmosphere (MA) storage alleviated CI by facilitating the accumulation of jasmonic acids (JAs) and γ-aminobutyric acid (GABA) in ‘Zhonghuashoutao’ (‘ZHST’) peach fruit. Here we show that 10% CO_2_ alone can improve cold tolerance, with ethylene response factor 17 (PpERF17) identified as a pivotal transcription factor (TF) that promotes biosynthesis of JAs and GABA. Stable transformation of *PpERF17* in tobacco resulted in reduced cold damage, attributed to decreased levels of hydrogen peroxide (H_2_O_2_) and malondialdehyde (MDA), as well as enhanced accumulation of JAs and GABA. Moreover, under eCO_2_, PpMYC2.1, the master regulator of JA signaling, was found to activate transcription of *13S-lipoxygenase* (*Pp13S-LOX*), *allene oxide synthase* (*PpAOS*), *12-oxophytodienoate reductase 3* (*PpOPR3*), and *glutamate decarboxylase* (*PpGAD*), while also inducing the expression of the upstream TF *PpERF17*, thereby establishing positive feedback loops upregulating JA and GABA biosynthesis. Finally, application of methyl jasmonate (MeJA) to fruit before shelf transfer from cold storage alleviated chilling injury development, due to increased accumulation of JAs and GABA as a result of raised expression of related biosynthetic genes. Collectively, our results suggest that eCO_2_-induced *PpERF17* enhances JAs and GABA accumulation while activating the JA signaling pathway. This contributes to a positive feedback loop mediated by PpMYC2.1, ultimately alleviating CI of peach fruit through the sustained accumulation of JAs and GABA.

## Introduction

Low-temperature storage has become a widely adopted practice to prolong the postharvest lifespan of many fruits, including peach (*Prunus persica* (L.) Batsch). However, the onset of chilling injury (CI) during protracted storage periods at inappropriate temperatures may negate the beneficial effects on fruit quality and even accelerate their spoilage. Internal browning (IB) is one of the most distinctive symptoms of CI emerging during the shelf life of the fruit following cold storage, and this significantly compromises fruit quality and reduces its commercial value [1]. This has stimulated the exploration of biotechnological strategies, including treatments with methyl jasmonate (MeJA) and γ-aminobutyric acid (GABA), as well as controlled atmosphere (CA) storage, aimed at enhancing chilling tolerance, yielding substantial insights into the effect of low temperatures on modulation of energy metabolism, reactive oxygen species (ROS) production and scavenging, and membrane stability changes, which has improved our understanding of the intricate mechanisms that underlie CI [2–4].

Exogenous MeJA and GABA, which serve respectively as a phytohormone and an osmolyte, have been utilized to delay the occurrence of CI in peach fruit [5, 6]. We found that enhanced cold tolerance was associated with the accumulation of endogenous GABA and jasmonic acid (JA) [7]. Glutamate decarboxylase (GAD) catalyzes the irreversible decarboxylation of glutamate to produce GABA [6]. The biosynthetic pathway of JA encompasses a series of enzymes, including 13S-lipoxygenase (13S-LOX), allene oxide synthase (AOS), allene oxide cyclase (AOC), and 12-oxophytodienoate reductase 3 (OPR3). The bioactive derivative of JA, jasmonoyl-isoleucine (JA-Ile), is synthesized by jasmonic acid-amino synthetase (JAR) and is perceived by the F-box protein CORONATINE INSENSITIVE1 (COI1). This interaction leads to the degradation of jasmonate ZIM-domain proteins (JAZ), ultimately resulting in the release of MYC2, a key regulator of JA signaling [8]. Notably, it has been reported that exogenous applications of GABA and JA can mutually promote their endogenous synthesis in tomato, loquat, and strawberry [9–11]. However, the molecular mechanisms underlying the synergistic enhancement of cold tolerance through the possible interplay between JAs and GABA remain unclear. 

CA storage, which precisely regulates O_2_ and CO_2_ levels within storage environments, is both eco-friendly and health-oriented, as it eliminates the need for exogenous chemical compounds to prolong the shelf life and combats diseases of postharvest fruits and vegetables [12]. Recent studies have demonstrated that high CO₂ levels alone hold significant potential in mitigating quality deterioration in various fruits, such as grapes and strawberries [13–15]. For instance, it has been established that high CO₂ levels positively regulate the preservation of grape pulp and rachis through the modulation of pathogenesis-related proteins (PRs), consequently delaying water loss and inhibiting fungal decay during postharvest cold storage [14]. In the case of peach fruit, despite the extensive application of CA storage methods to prolong fruit storage life, it is unclear whether both oxygen deficiency and elevated CO_2_ (eCO_2_) levels are essential for alleviation of CI.

**Fig. 1:**
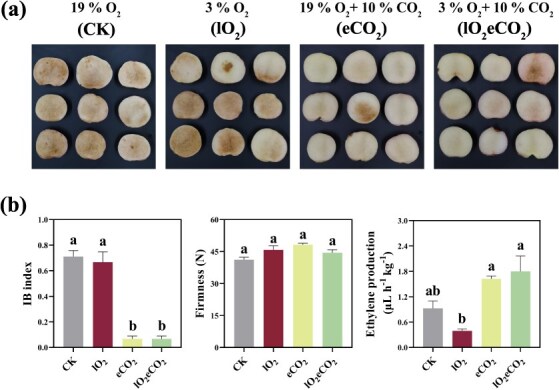
**Physiological changes in ‘ZHST’ peach fruit after 3 days at 20°C following 49 days 0°C storage with low O**_**2**_  **and/or eCO**_**2**_**.** (a) Pictures showing IB after 3 days at 20°C. (b) Profiles of IB index, flesh firmness, and ethylene production. Different lowercase letters denoted a significant difference (*p* < 0.05). CK, 19% O_2_; eCO_2_, 19% O_2_ + 10% CO_2_; lO_2_, 3% O_2_; lO_2_eCO_2_, 3% O_2_ + 10% CO_2_.

Increasing evidence indicates that transcription factors (TFs) are instrumental in mediating responses to eCO_2_ condition, thus modulating stress tolerance. This is exemplified by the involvement of tomato *ethylene response factor 1* (*SlERF1*) and *heat shock transcription factor A2* (*SlHSFA2*), *LtWRKY21*, as well as *SlNAC43*, in eCO_2_-mediated alleviation of heat stress [16], drought and water deficit [17], and pathogen attack [18], respectively. TFs governing the fruit responses to eCO_2_ levels have also been characterized. Collectively, they play a crucial role in postdeastringency softening and in retarding fruit water loss, including ERFs, WRKYs, NACs, and MYBs in persimmon [19–21] as well as ERFs and WRKYs in grape [14, 22]. In addition, MYC2, a basic helix–loop–helix (bHLH) TF recognized as a master regulator of JA signaling, also plays a beneficial role in plant cold resistance, as reported in banana [23], tomato [8], trifoliate orange [24], and grape [25]. Recent studies underscore the importance of TFs as crucial regulators implicated in the development of physiological disorders such as CI [26, 27]. However, the possible role of MYC2 in GABA biosynthesis and its potential activation by eCO_2_ in the regulation of cold response in peach fruit remain to be elucidated.

In a prior work, we identified PpERF61 as a central TF crucial for overcoming cold stress in response to modified atmosphere (MA) treatment [7]. However, the effectiveness of eCO_2_ alone in alleviating CI remains ambiguous, and the regulatory network involving TFs related to JA and GABA biosynthesis in peach fruit response to eCO_2_ is not well understood. Herein, we show that eCO_2_ alone, rather than O_2_ deficiency, is sufficient to significantly mitigate the CI of ‘Zhonghuashoutao’ (‘ZHST’) peach fruit. eCO_2_ induced another ERF, PpERF17, which directly activated *Pp13S-LOX*, *PpAOS*, *PpOPR3*, and *PpGAD* to regulate endogenous JAs and GABA accumulation, subsequently activating the JA signaling component PpMYC2.1, which further reinforces this pathway. Taken together, this work reveals a positive feedback loop that operates to upregulate JA biosynthesis, mediated by PpERF17 and PpMYC2.1, and provides evidence for a novel molecular mechanism for regulating GABA in peach fruit through the PpMYC2.1–*PpGAD* pathway induced by eCO_2_ in a low-temperature environment.

## Results

### Elevated CO_2_ level rather than low O_2_ concentration alleviates IB occurrence in peach fruit after transfer to shelf conditions following cold storage

Previously we demonstrated that MA treatment mitigated peach CI during ripening following cold storage [7]. To further investigate individual effect of low O_2_ and eCO_2_ treatment on alleviation of peach CI, ‘ZHST’ peach fruit were subjected to treatments of 19% O_2_ with negligible CO_2_ (<0.05%) (CK), 3% O_2_ with negligible CO_2_ <0.01% (lO_2_, short for low O_2_), 19% O_2_ + 10% CO_2_ (eCO_2_), and 3% O_2_ + 10% CO_2_ (lO_2_eCO_2_). All treated fruits were stored for 49 days at 0°C and then transferred to shelf conditions at 20°C for 3 days. Notably, eCO_2_ alone was sufficient to suppress CI after 3 days of shelf storage in ‘ZHST’, as evidenced by a significant reduction in the IB index, whereas lO_2_ alone did not produce a significant effect (Fig. 1a and b). It was also observed that lO_2_ reduced ethylene production during the shelf life period, but this could be restored by eCO_2_ (Fig. 1b). Furthermore, flesh firmness was unaffected by either lO_2_ or eCO_2_. Since eCO_2_ alone could effectively mitigate CI occurrence in ‘ZHST’ peach fruit, only CK and eCO_2_ samples were included in subsequent analysis.

### Elevated CO_2_ level regulates JAs and GABA synthesis potentially by stimulating the expression of TF *PpERF17*

**Fig. 2:**
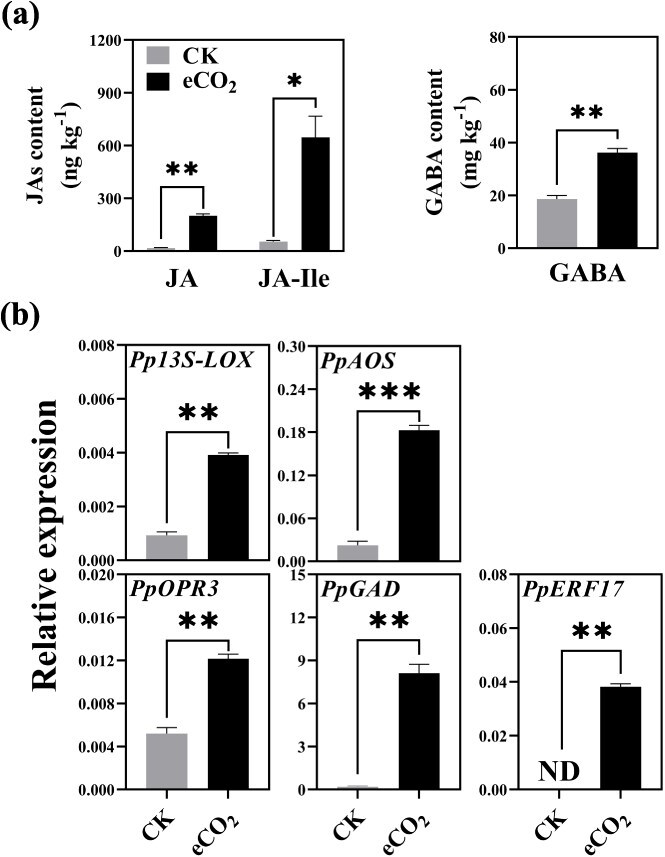
**Effect of eCO**_**2**_  **treatment on the metabolic pathways of JA and γ-aminobutyric acid (GABA) in ‘ZHST’ peach flesh after 0°C storage for 49 days.** (a) Contents of JA, JA-Ile, and GABA. (b) Expression of JA biosynthetic genes, GABA biosynthetic gene, and TF gene *PpERF17*. Student’s *t*-test with significance levels indicated as follows: ^*^ for *p* < 0.05, ^**^ for *p* < 0.01, and ^***^ for *p* < 0.001. 13S-LOX, 13S-lipoxygenase; AOS, allene oxide synthase; CK, 19% O_2_; eCO_2_, 19% O_2_ + 10% CO_2_; ERF, ethylene response factor; GAD, glutamate decarboxylase; ND, not detectable; OPR3, 12-oxophytodienoate reductase 3.

In our prior work, the inhibition of peach IB was attributed to accumulation of JAs and GABA following MA treatment [7]. Herein, we measured JA, JA-Ile, and GABA contents in flesh tissue and observed that the accumulation of these compounds was strongly induced by the eCO_2_ atmosphere, with increases of 10.8-fold, 10.9-fold, and 0.9-fold, respectively (Fig. 2a). Consistent with these findings, the expression levels of JA biosynthetic genes *Pp13S-LOX*, *PpAOS*, and *PpOPR3* and GABA biosynthetic gene *PpGAD* were significantly higher in response to eCO_2_ treatment (Fig. 2b).

To explore upstream regulator(s) implicated in the stimulation of JAs and GABA synthesis, we conducted a *cis*-motif analysis using the candidate TFs identified in our previous study [7]. PpERF17 (Prupe.7G194400) is the only TF predicted to simultaneously bind to promoters of *Pp13S-LOX*, *PpAOS*, *PpOPR3*, and *PpGAD* (Table S1). Meanwhile, correlation analysis indicated that PpERF17 could possibly function as a novel activator for facilitating JAs and GABA accumulation, as evidenced by a significantly high correlation between the expression of *PpERF17* and those of the relevant structural genes (Fig. S1). It was also observed that *PpERF17* was not detectable in CK fruit but was significantly induced by eCO_2_ (Fig. 2b), indicating that *PpERF17* responded to eCO_2_ level and potentially triggered JAs and GABA accumulation, ultimately alleviating peach CI.

### The activation effect of PpERF17 on the transcription of JAs and GABA biosynthetic genes

**Fig. 3:**
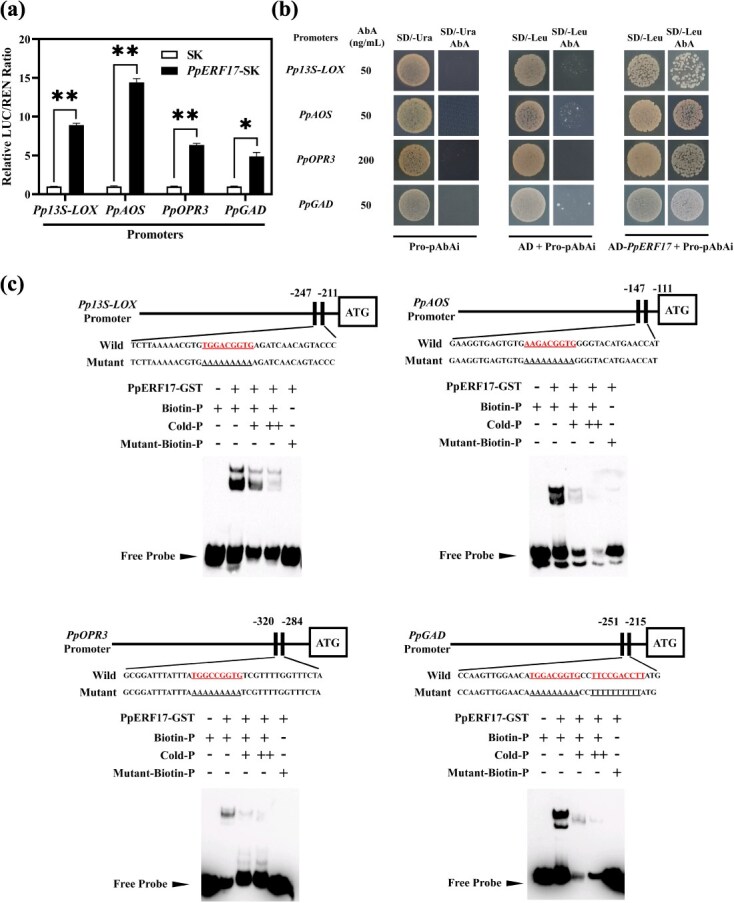
**Interaction of PpERF17 with the promoters of JA and GABA biosynthetic genes *Pp13S-LOX*, *PpAOS*, *PpOPR3*, and *PpGAD*.** (a) Dual-luciferase assay. (b) Y1H assay analysis. (c) EMSA. ‘−’ and ‘+’ indicate absence and presence of the probe or protein, respectively. ‘+’ and ‘++’ indicate 50-fold and 500-fold concentration of cold probes, respectively. Student’s *t*-test with significance levels indicated as follows: ^*^ for *p* < 0.05, ^**^ for *p* < 0.01, and ^***^ for *p* < 0.001. 13S-LOX, 13S-lipoxygenase; AbA, aureobasidin A; AD, pGADT7 vector; AOS, allene oxide synthase; ERF, ethylene response factor; GAD, glutamate decarboxylase; GST, glutathione *S*-transferase; LUC, firefly luciferase; OPR3, 12-oxophytodienoate reductase 3; REN, renilla luciferase; SD, synthetic dropout medium.

PpERF17 is characterized as an APETALA2 (AP2)/ERF TF consisting of 215 amino acids (Fig. S2). It is present in both the nucleus and plasma membrane as revealed by subcellular localization analysis (Fig. S3). To investigate whether PpERF17 mediated the accumulation of JAs and GABA via activating the expression of JAs and GABA synthesis-related genes directly, dual-luciferase assays were performed and the results indicated that PpERF17 strongly activated the expression of *Pp13S-LOX*, *PpAOS*, *PpOPR3*, and *PpGAD*, with increases of 7.9-fold, 13.4-fold, 5.3-fold, and 3.9-fold, respectively (Fig. 3a). For yeast one-hybrid (Y1H) assays, the promoters, consisting of fragments from 1000, 2000, 2000, and 2000 bp upstream of the coding sequence (CDS), respectively, for *Pp13S-LOX*, *PpAOS*, *PpOPR3*, and *PpGAD*, were cloned into pAbAi vector. Results revealed that PpERF17 could physically bind to the promoters of these genes (Fig. 3b). Electrophoretic mobility shift assay (EMSA) further confirmed these interactions, with binding signals observed when PpERF17 was combined with promoters of related genes, while these signals diminished with the increasing concentration of cold probes as competitors and completely disappeared when the biotin probes were mutated (Fig. 3c). Collectively, these findings demonstrated that PpERF17 could directly activate JA and GABA biosynthesis in peach fruit.

### Functional characterization of PpERF17 in peach fruit and tobacco

**Fig. 4:**
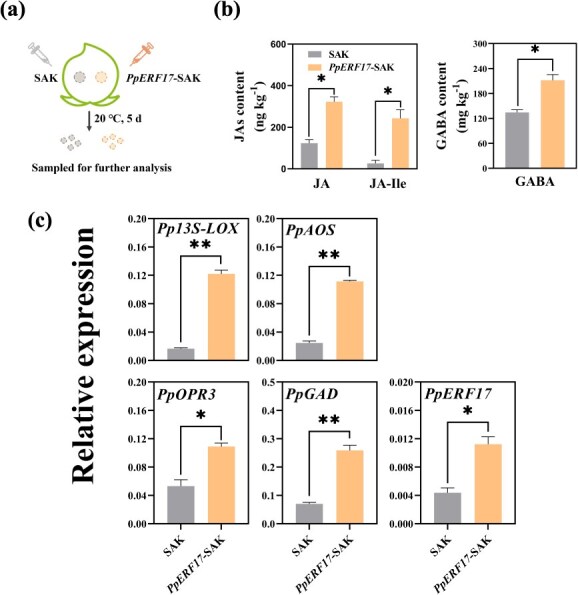
**Effects of transient overexpression of *PpERF17* on JA and GABA synthesis in peach fruit.** (a) Schematic diagram for transient overexpression in peach fruit. (b) JAs and GABA contents of the injected flesh in empty SAK vector and *PpERF17*-SAK-infiltrated peach fruit. (c) Expression of JAs and GABA synthesis-related genes and *PpERF17* in empty SAK vector and *PpERF17*-SAK-infiltrated peach fruit. Student’s *t*-test with significance levels indicated as follows: ^*^ for *p* < 0.05, ^**^ for *p* < 0.01, and ^***^ for *p* < 0.001. 13S-LOX, 13S-lipoxygenase; AOS, allene oxide synthase; ERF, ethylene response factor; GAD, glutamate decarboxylase; OPR3, 12-oxophytodienoate reductase 3; SAK, pSAK277 vector.

Transient overexpression of *PpERF17* in peach fruit was conducted to elucidate its role in the regulation JAs and GABA accumulation (Fig. 4a). In comparison to SAK (an empty vector) fruit, JAs and GABA accumulation was markedly increased in *PpERF17*-SAK fruit (Fig. 4b), with the cells adjacent to the injection site displaying a considerably elevated expression levels of *PpERF17* as well as JAs and GABA biosynthetic genes (Fig. 4c).

**Fig. 5:**
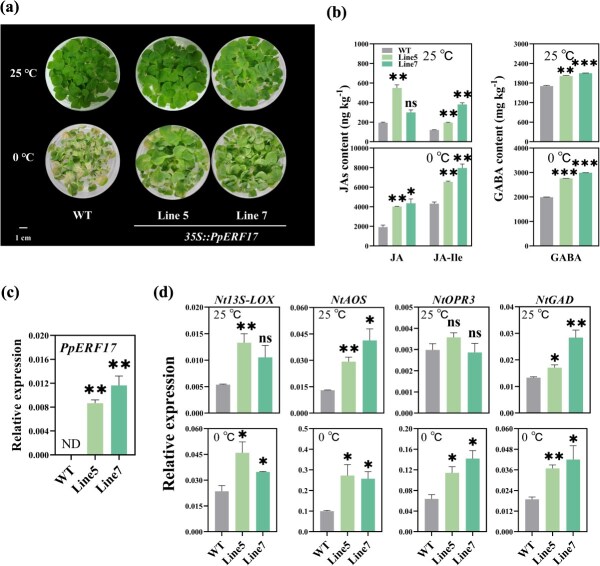
**Cold tolerance assessment of transgenic tobacco (*Nicotiana tabacum*) plants overexpressing *PpERF17*.** (a) Phenotypes of 21-day-old tobacco plants subjected to a cold treatment of 0°C for 14 days. (b) JA, JA-Ile, and GABA contents. (c) Expression of *PpERF17*. (d) Expression of JA and GABA biosynthetic genes. Student’s *t*-test with significance levels indicated as follows: ^*^ for *p* < 0.05, ^**^ for *p* < 0.01, and ^***^ for *p* < 0.001. 13S-LOX, 13S-lipoxygenase; AOS, allene oxide synthase; ERF, ethylene response factor; GABA, γ-aminobutyric acid; GAD, glutamate decarboxylase; JA, jasmonic acid; ns, not significant; OPR3, 12-oxophytodienoate reductase 3; WT, wild type.

Stable transformation of *PpERF17* in tobacco corroborated its functional role. A significant increase of cold tolerance was observed in transgenic tobacco lines overexpressing *PpERF17* (Fig. 5a). Wild-type (WT) plants exhibited wilting and yellowing when subjected to a cold stress treatment of 0°C for 14 days, whereas both transgenic lines were less affected. Significantly lower levels of hydrogen peroxide (H_2_O_2_) and malondialdehyde (MDA) were observed in the *PpERF17*-OE plants, compared with WT, under cold stress (Fig. S4). The contents of JAs and GABA in transgenic tobacco plants were higher than those in WT at both room and low temperature, with the difference being particularly pronounced under low temperature (Fig. 5b). The expression of *PpERF17* was confirmed in the *PpERF17*-OE plants (Fig. 5c), and the expression levels of *Nt13S-LOX*, *NtAOS*, *NtOPR3*, and *NtGAD* were upregulated in transgenic tobacco leaves (Fig. 5d). Together, these results support the conclusion that *PpERF17* functions as a positive regulator of cold tolerance by upregulating genes for JAs and GABA biosynthesis.

### PpMYC2.1 has a positive feedback effect on transcription of JAs and GABA biosynthesis genes

**Fig. 6:**
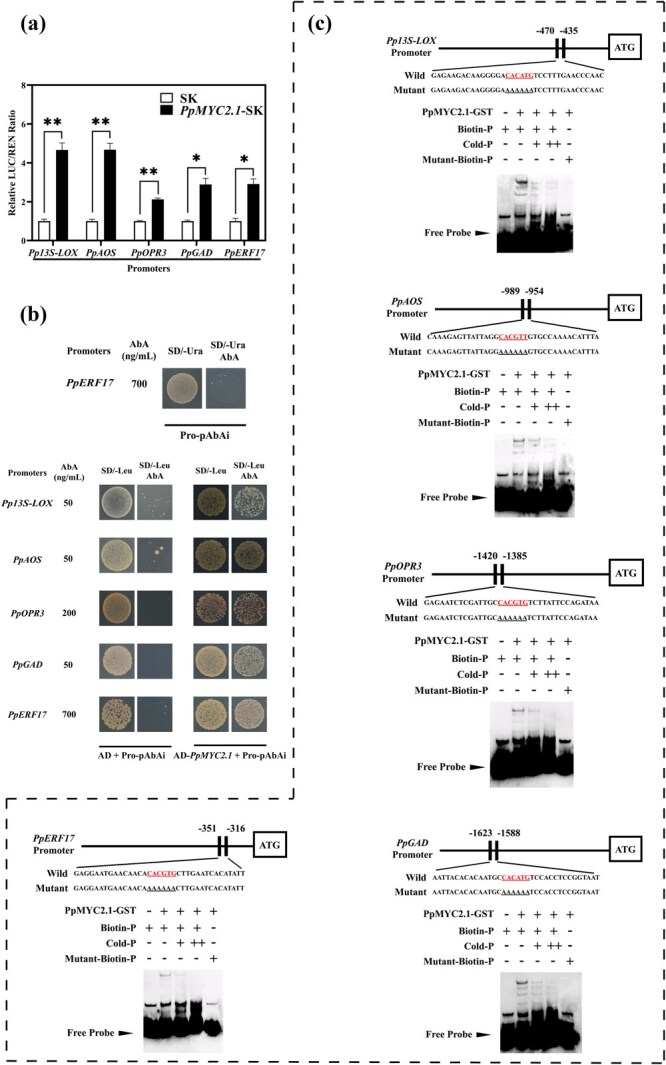
**Interaction of PpMYC2.1 with the promoters of JA and GABA biosynthetic genes and *PpERF17*.** (a) Dual-luciferase assay. (b) Y1H assay. (c) EMSA. ‘−’ and ‘+’ indicate absence and presence of the probe or protein, respectively. ‘+’ and ‘++’ indicate 20-fold and 200-fold concentration of cold probes, respectively. Student’s *t*-test with significance levels indicated as follows: ^*^ for *p* < 0.05, ^**^ for *p* < 0.01 and ^***^ for *p* < 0.001. 13S-LOX, 13S-lipoxygenase; AbA, aureobasidin A; AD, pGADT7; AOS, allene oxide synthase; ERF, ethylene response factor; GAD, glutamate decarboxylase; GST, glutathione *S*-transferase; LUC, firefly luciferase; OPR3, 12-oxophytodienoate reductase 3; REN, renilla luciferase; SD, synthetic dropout medium.

MYC2.1 functions as a master regulator in the JA signaling pathway. Previously we observed that *PpMYC2.1* (Prupe.5G035400) was significantly upregulated in ‘ZHST’ peach fruit in response to MA treatment [7]. In this study, we further investigated its expression pattern under eCO_2_ treatment to explore its possible role in regulation of JAs and GABA accumulation. Reverse-transcription quantitative polymerase chain reaction (RT-qPCR) revealed that *PpMYC2.1* was remarkably induced both under eCO_2_ treatment and following transient overexpression of *PpERF17* in peach fruit (Fig. S5a and b). PpMYC2.1 was localized in the nucleus as revealed by subcellular localization analysis (Fig. S3). Subsequently, dual-luciferase assays indicated that PpMYC2.1 regulated the transcription of both JA and GABA biosynthetic genes as well as *PpERF17* (Fig. 6a). The binding of PpMYC2.1 to the promoters of the above-mentioned genes was also further validated by Y1H assay. Yeast cells harboring PpMYC2.1-pGADT7 and pro-pAbAi successfully grew on the synthetic dropout medium lacking Leu (SD/−Leu) supplemented with aureobasidin A (AbA), whereas coexpression pro-pAbAi with the empty pGADT7 plasmid did not support growth (Fig. 6b). The result of EMSA also indicated that the recombinant PpMYC2.1 protein could interact with the promoters of *Pp13S-LOX*, *PpAOS*, *PpOPR3*, *PpGAD*, and *PpERF17* (Fig. 6c). These findings suggested that PpMYC2.1 served as a direct transcriptional activator of JAs and GABA synthesis genes. Furthermore, PpMYC2.1 induced the expression of upstream regulator *PpERF17* upon activation of the JA signaling pathway, resulting in a substantial accumulation of JAs and GABA, thereby alleviating CI in peach fruit.

### Prior MeJA treatment effectively alleviates CI of peach fruit subsequently transferred to shelf conditions at 20°C

**Fig. 7:**
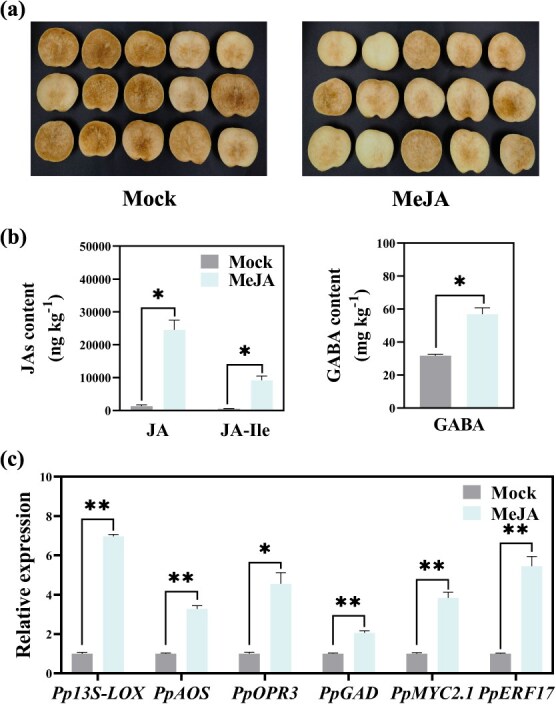
**Effects of MeJA treatment on regulating CI symptoms in peach fruit.** (a) Pictures of peach fruit taken at the end of 3-day shelf life at 20°C after storage at 5°C for 28 days. For MeJA treatment, the fruit were immersed in 1 mM solution for 10 min just prior to shelf transfer. (b) Changes in JA and GABA contents of peach after MeJA treatment. (c) Expression of *PpERF17* and *PpMYC2.1* and related structural genes in peach fruit following MeJA treatment. Student’s *t*-test with significance levels indicated as follows: ^*^ for *p* < 0.05, ^**^ for *p* < 0.01, and ^***^ for *p* < 0.001. 13S-LOX, 13S-lipoxygenase; AOS, allene oxide synthase; ERF, ethylene response factor; GAD, glutamate decarboxylase; OPR3, 12-oxophytodienoate reductase 3.

To assess the effect of JA on the alleviation of peach CI and to investigate whether endogenous GABA biosynthesis could be modulated by JA signaling, we applied MeJA treatment (1 mM for 10 min) at the termination of cold storage and subsequently transferred the fruit to 20°C to simulate commercial shelf life. As anticipated, MeJA treatment effectively mitigated the manifestation of CI of peach fruit (Fig. 7a). This supports the conclusion that the accumulation of JAs during storage is beneficial in repressing CI development during the subsequent shelf life, as evidenced by a significantly lower IB index and reduced levels of H_2_O_2_ (Fig. S6a and b). Furthermore, endogenous JAs and GABA increased in accordance with the elevated expression levels of JA and GABA biosynthetic genes following MeJA treatment (Fig. 7b and c). Interestingly, the expression levels of *PpMYC2.1* and *PpERF17* were also significantly induced in response to exogenous MeJA treatment (Fig. 7c), implying the operation of the positive feedback loop that plays a crucial role in alleviating the manifestation of CI in peach fruit.

## Discussion

Plants are vulnerable to various abiotic stresses, which ultimately lead to detrimental impacts on agricultural productivity and food security [28]. In a number of situations, it has been found that elevated atmosphere CO_2_ is helpful for raising tolerance to stresses such as high temperature and drought, involving the participation of phytohormones, such as JA, and their associated signaling pathways, ROS, osmolytes like GABA, and other factors [16, 29–32]. Interestingly, during diverse plant–parasite interactions, it has been reported that eCO_2_ negatively regulates JA signaling in tea [33], rice [34], and tomato [35], while it can exhibit positive effects in tobacco by increasing JA levels and the accumulation of defense-related secondary metabolites [34]. Conversely, GABA tends to accumulate universally under eCO_2_ in fruits including tomato [36], fresh-cut pear [37], and strawberry [38].

Although MA and CA storage have been widely applied to delay fruit senescence and to restrain pathogen attack, the beneficial effects of eCO_2_ and the underlying mechanisms in the postharvest research area are, however, far from being well understood. The problem of CI in peach is most noticeable and affects their shelf life and consumer acceptability after transfer of fruit from cold storage to room temperatures. In our previous study, we demonstrated that the levels of endogenous JAs and GABA exhibited a negative correlation with the severity of IB and contributed to the mitigation of CI under MA treatment in both ‘Jinxiu’ and ‘Zhonghuashoutao’ peach fruit [7]. In this study, we show that eCO_2_, rather than oxygen deficiency, effectively mitigated the detrimental impacts and alleviated CI in peach subjected to prolonged cold storage, potentially attributable to a synergistic effect between eCO_2_ and low temperature (Fig. 1a). It was found that enhanced accumulation of JA, JA-Ile, and GABA was clearly associated with a reduction in CI (Fig. 2a). The effectiveness of JAs and GABA in mediating plant chilling tolerance has also been recognized in other plants such as apple, grape, and tomato [39–42]. Specifically, JA and GABA improved apple cold tolerance by modulating the CBF pathway and enhancing antioxidant capacity, respectively [39, 41].

Plants control their gene transcriptional response to external stimuli such as cold stress through a series of TFs that activate transcription of specific target genes [43]. To date, many regulators have been identified as being involved in transcriptional processes in the regulation of JA and GABA biosynthesis in response to abiotic stress in plants [39, 41]. In the case of JA synthesis, members of the AP2/ERFs (APETALA2/ethylene-responsive factors) superfamily, such as AtERF018 [44], SlERF15 and SlERF16 [45], BrERF72 [46], and MaERF10 [47] are involved in this process. In GABA biosynthesis, *GAD* was positively regulated by MdCBF3 [39] and negatively regulated by SlTHM27 [48]. In this study, our results showed that both structural genes of JA and GABA biosynthesis were significantly activated by PpERF17, indicating its important role as an upstream regulator of the cold stress response (Fig. 2). Transient overexpression of *PpERF17* in peach fruit further confirmed its regulatory effects on JA and GABA accumulation (Fig. 4). Additionally, ectopic overexpression of *PpERF17* enhanced tobacco cold tolerance and lowered MDA and H_2_O_2_ levels (Fig. S4). The transcript levels of *Nt13S-LOX*, *NtAOS*, *NtOPR3*, and *NtGAD* as well as JAs and GABA contents in *PpERF17*-OE plants were significantly increased under low-temperature conditions (Fig. 5). These findings, together with our previous observations on MA-induced mitigation of peach CI, indicate that JA and GABA are core components in the peach fruit comprehensive chilling response processes and TFs like PpERF17 are important regulators in the associated transcriptional cascade underlying the plant cold response.

MYC2, the bHLH protein, and a core element in JA signaling, activates downstream JA-responsive genes by specifically recognizing G-box motifs within their promoters [8]. For example, PtrMYC2 directly binds to the G-box *cis*-elements in the promoter of betaine aldehyde dehydrogenase (PtrBADH-l) to modulate glycine betaine (GB) accumulation, thus improving cold tolerance [24]. Increasing evidence suggests that a MYC2-mediated positive feedback loop orchestrates plant defense response such as osmotic and cold stress tolerance [8, 49] and herbivore resistance [45] as well as plant development process such as diurnal flower-opening time of rice [50]. However, MYC2 has also been shown to regulate repression of JA signaling in tomato and rice via the negative feedback loop [51, 52]. Our findings suggest that PpMYC2.1 could respond to both eCO_2_ and increased endogenous JAs level, as evidenced by its upregulation following eCO_2_ and MeJA treatment (Fig. 7c; Fig. S5). Transient overexpression of *PpERF17* in peach fruit also induced the accumulation of transcripts of *PpMYC2.1*, suggesting the potential involvement of PpMYC2.1 in the accumulation of endogenous JAs (Fig. 4). We have provided molecular evidence that PpMYC2.1 transcriptionally regulates GABA biosynthetic gene *PpGAD* (Fig. 6). Additionally, MeJA treatment was found to promote accumulation of both JAs and GABA in fruit, along with the elevated expression of TF *PpMYC2.1* and structural gene *PpGAD* (Fig. 7). This indicates a mechanistic interaction between JA and GABA, which contributes to the alleviation of CI in peach fruit through enhanced accumulation of JA and GABA, ultimately extending fruit shelf life. On the other hand, dual-luciferase assay, Y1H, and EMSA substantiated the interaction between PpMYC2.1 and the promoters of *Pp13S-LOX*, *PpAOS*, *PpOPR3*, and the upstream TF *PpERF17* (Fig. 6). These findings demonstrated the existence of two distinct positive feedback loops: one that encompasses the PpMYC2.1-mediated activation of JA biosynthesis structural genes, and a second that forms a regulatory cascade involving PpMYC2.1–PpERF17–JA biosynthesis.

**Fig. 8:**
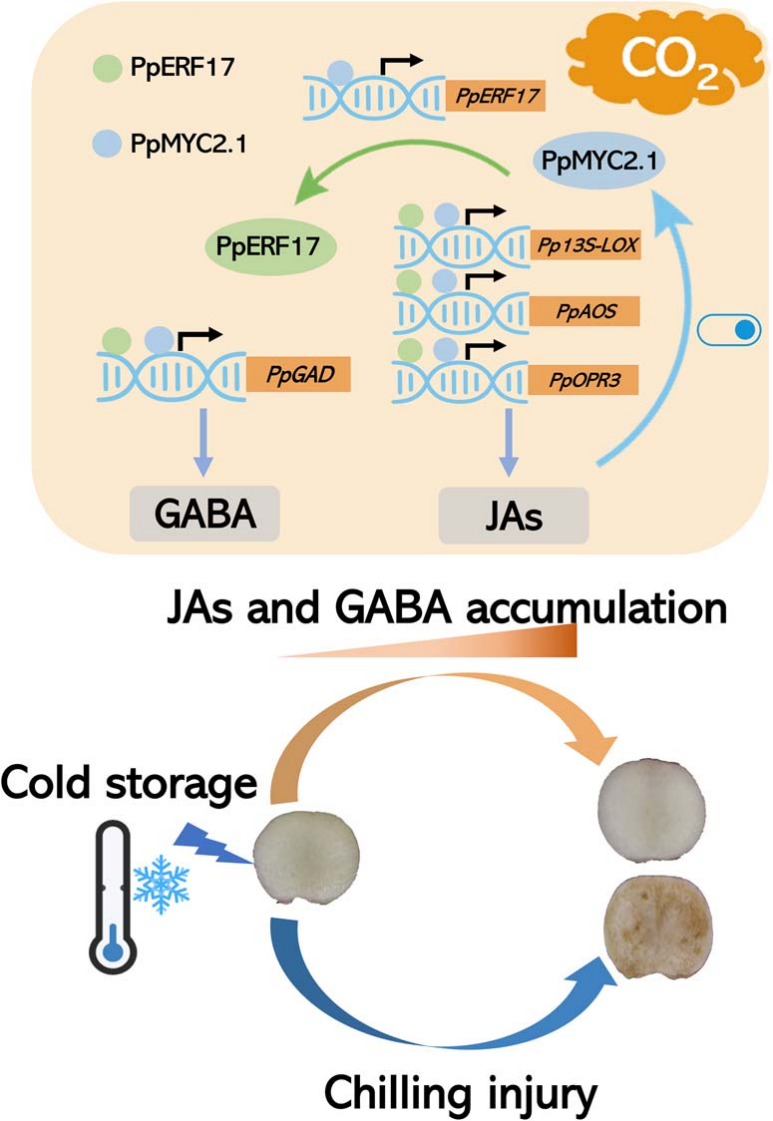
A proposed model for the role of PpERF17 and PpMYC2.1 in JA- and GABA-mediated alleviation of postharvest CI under eCO_2_ in peach fruit.

Based on the findings of this study, we propose a model of the roles of PpERF17 and PpMYC2.1 in mediating cold tolerance in peach fruit under eCO_2_ conditions (Fig. 8). The expression of *PpERF17*, induced by eCO_2_, stimulates the expression levels of *Pp13S-LOX*, *PpAOS*, *PpOPR3*, and *PpGAD*, thereby initiating the accumulation of JAs and GABA as well as activating JA signaling. The accumulation of JAs is reinforced through the transcriptional regulatory cascades involving PpMYC2.1–JA and PpMYC2.1–PpERF17–JA, while GABA accumulation is enhanced by the PpMYC2.1–*PpGAD* module. This develops a positive feedback loop responsible for massive accumulation of JAs and GABA and ultimately improving cold tolerance of peach fruit (Fig. 8). Thus, eCO_2_ treatment represents a viable chemical-free alternative for extending the storage life of peach fruit, as opposed to the exogenous application of JA and GABA.

Recent research has indicated that mitogen-activated protein kinase 4 (MPK4) may function as a CO_2_ receptor in plants [53], and that the phosphorylation of MdERF17 by MdMPK4 plays a crucial role in modulating fruit chlorophyll degradation during peel coloration [54]. It would be valuable to explore whether PpMPK4 serves as a link between eCO_2_ sensing and increased transcription level of *PpERF17* and its target genes. Research into the underlying mechanisms by which eCO_2_ enhances fruit adaptability to postharvest cold environments is currently under the way.

## Materials and methods

### Plant materials and treatment

Peach (*P. persica*) cv. ‘Zhonghuashoutao (ZHST)’ at commercial maturity were collected from an orchard in Shandong Province. Fruit, in uniform maturity and size, free from disease symptom or mechanical injury were subjected to precooling for 12 h at 0°C. After that, the fruit were sorted randomly into four groups: 3% O_2_ + 97% N_2_, 3% O_2_ + 10% CO_2_ + 87% N_2_, 19% O_2_ + 81% N_2_, and 19% O_2_ + 10% CO_2_ + 71% N_2_. For simplicity, these groups were named as low O_2_ (lO_2_), low O_2_ plus elevated CO_2_ (lO_2_eCO_2_), ambient atmosphere control (CK), and elevated CO_2_ (eCO_2_), respectively. Each group of fruit was sealed in a 21-l container flowed with designed gas compositions and stored at 0°C and 85%–90% relative humidity for 49 days. After storage, all fruit were transferred to 20°C for 3 days shelf life.

For MeJA treatment, ‘ZHST’ peach fruit were stored at 5°C and 85%–90% relative humidity for 28 days. After this period, fruit were immersed in 1 mM MeJA (Sigma-Aldrich, St. Louis, MO, USA) solution for 10 min. The mock group fruit were soaked for 10 min in deionized water supplemented with corresponding amount of ethanol as used in preparation of MeJA solution. Both the MeJA-treated fruit and the mock fruit were subsequently put at 20°C to test 3 days shelf life.

Three biological replicates, with three fruit for each replicate, were set for each sampling point. At the termination of cold storage and shelf life, fruit were collected and the mesocarp was cut, quickly frozen with liquid nitrogen, and stored at −80°C.

### Determination of physiological traits

The determination of all physiological traits was conducted in accordance with Zhu *et al.* [55]. The rate of ethylene production was measured using gas chromatography (Agilent Technologies 7890B GC System, Santa Clara, CA, USA). The IB index was assessed based on the browning degree of the flesh. Firmness was determined after removing a 1-mm-thick slice of skin from opposite sides at the equator of each fruit.

### Measurement of H_2_O_2_ and MDA

H_2_O_2_ was extracted using a commercial H_2_O_2_ assay kit (Solarbio Science and Technology Co., Ltd., Beijing, China). The reaction between H_2_O_2_ and titanium sulfate yields a yellow product, exhibiting a maximal absorption peak at 415 nm. The H₂O₂ content was determined and calculated according to the manufacturer’s instructions.

MDA was extracted using the 100 g/l trichloroacetic acid solution [56]. Under acidic and high-temperature conditions, MDA reacts with thiobarbituric acid to form a reddish-brown product, exhibiting a maximum absorption peak at 532 nm. The absorbances at 450, 532, and 600 nm were determined using a UV spectrophotometer and the MDA content was calculated with the formula as described by Wang *et al.* [57].

### Analysis of JA, JA-Ile, and GABA

The extraction and analysis of JA, JA-Ile, and GABA were performed according to protocols established in our previous study [7, 58]. In brief, JA and JA-Ile were extracted using the prechilled extraction solvent (isopropanol:H_2_O: concentrated hydrochloric acid = 2:1:0.005, v/v/v), along with 10 ng of internal standards (D6-JA, Q/C/C, Newark, USA; D6-JA-Ile, Q/C/C, Newark, USA). JA and JA-Ile contents were determined by the Agilent 6460 Triple Quadrupole LC/MS/MS system (Agilent Technologies, Santa Clara, CA, USA) and quantified by comparing with the corresponding internal standards. GABA was determined after Berthelot colorimetric reaction with products monitored at 645 nm, and the standard curve was used to analyze GABA contents.

### DNA, RNA extraction, and RT-qPCR analysis

Genomic DNA and total RNA of peach were extracted from leaves and fruit, respectively, following our previous methods [59]. Total RNA was isolated from tobacco leaves using RNA Easy Fast Plant Tissue Kit (TIANGEN, Beijing, China). Plant gDNA removal and cDNA synthesis were carried out according to the manufacturer’s instruction using HiScript® II Q RT SuperMix for qPCR (+gDNA wiper) (Vazyme, Nanjing, China). RT-qPCR was performed using ChamQ Universal SYBR qPCR Master Mix (Vazyme, Nanjing, China) on a CFX96 machine (Bio-Rad, CA, USA). *PpTEF2* (JQ732180) and *NtEF1α* (NM001326165) were utilized as internal reference genes to normalize gene expressions of peach and tobacco, respectively. Primers utilized in RT-qPCR analysis are detailed in Table S2.

### *Cis*-motif analysis, multiple protein sequence alignments, and vector construction

PlantRegMap was used to identify potential binding site motifs (https://plantregmap.gao-lab.org/). The presented information included only the TFs and their respective regulatory target genes that were predicted to have binding sites within this database. Multiple protein sequence alignments were carried out using DNAMAN 6.0. Primers used for gene cloning and vector construction are provided in Table S3.

### Tobacco stable transformation and cold tolerance assessment

To generate transgenic plants, the full-length CDS of *PpERF1*7 was inserted into the vector pSAK277 and transformed into *Agrobacterium tumefaciens* strain EHA105. Tobacco stable transformation was performed in accordance with the leaf disc transformation method [60]. T1 transgenic plants were screened on selected medium containing kanamycin (100 mg/l) and kept in a growth room (16 h light and 8 h dark, 25°C). For cold stress treatment, 21-day-old transgenic tobacco (OE-5 and OE-7) and WT plants were kept at 0°C for 14 days and leaves were sampled for measurements of JA, JA-Ile, and GABA as well as RT-qPCR analysis.

### Transient overexpression in peach fruit

The *A. tumefaciens* strain EHA105 prepared for the tobacco stable transformation was also used for transient overexpression. The bacteria were grown at 28°C to an OD_600_ of 1.0 and resuspended in infiltration buffer (10 mM MES, pH 5.6; 10 mM MgCl_2_; 150 μM acetosyringone). The culture was injected into ‘Hanlumi’ peach fruit for transient overexpression, and the *A. tumefaciens* suspension containing empty pSAK277 vector was used as the negative control. The infiltrated fruits were placed at 20°C under 85%–90% relative humidity. The injected sites of flesh were sampled at 5 days after inoculation.

### Subcellular localization

CDS of *PpERF17* and *PpMYC2.1*, without the stop codon, were respectively inserted into pCAMBIA2300-eGFP vector and electroporated into *A. tumefaciens* strain GV3101*.* Transient expression and fluorescence detection were carried out using our previous protocols [59].

### Dual-luciferase assay

The CDS of *PpERF17* and *PpMYC2.1* were inserted into pGreenII 62-SK vectors and the promoter fragments of *Pp13S-LOX*, *PpAOS*, *PpOPR3*, *PpGAD*, and *PpERF17* were introduced into pGreenII 0800-LUC vectors [61]. Transient expression in *Nicotiana benthamiana* leaves was performed using *A. tumefaciens* strain GV3101. The dual-luciferase assay kit (Promega, Madison, WI, USA) was used to measure activities of firefly luciferase (LUC) and renilla luciferase (REN).

### Y1H assay

The promoter sequences of *Pp13S-LOX*, *PpAOS*, *PpOPR3*, *PpGAD*, and *PpERF17* were respectively inserted into pAbAi vector. The coding sequence of *PpERF17* and *PpMYC2.1* were inserted into pGADT7 vector. All the recombinant vectors were transformed into Y1HGold yeast strain. AbA was used to prevent self-activation and the interaction analysis was conducted as previously described [7].

### EMSA analysis

The full-length CDS of *PpERF17* and *PpMYC2.1* were cloned into pGEX-4 T-1 and recombinant vectors were expressed in *Escherichia coli* Rosetta (DE3). PpERF17-GST and PpMYC2.1-GST fusion proteins were induced by 300 μM β-D-1-thiogalactopyranoside (IPTG) at 16°C for 20 h and 500 μM IPTG at 16°C for 10 h, respectively. EMSA was carried out with the Chemiluminescent EMSA Kit (Beyotime, Shanghai, China). Probes used for EMSA are provided in Table S4.

### Statistical analysis

Means ± SE from three independent biological replicates are presented in this study. Data analysis was performed using SPSS 23.0 (IBM Corp., Armonk, N.Y., USA) by one-way analysis of variance (ANOVA) and Student’s *t*-test with significance levels indicated as follows: ^*^ for *p* < 0.05, ^**^ for *p* < 0.01, and ^***^ for *p* < 0.001. Figures were drawn using GraphPad Prism 9.0.2 (GraphPad Software, San Diego, CA, USA).

## Supplementary Material

Web_Material_uhaf014

## Data Availability

All data is available within manuscript and its supporting materials.

## References

[1] Ma YQ, Hu SQ, Chen GF. et al. Cold shock treatment alleviates chilling injury in peach fruit by regulating antioxidant capacity and membrane lipid metabolism. Food Qual Saf. 2022;6:1–11

[2] Albornoz K, Zhou JQ, Yu JW. et al. Dissecting postharvest chilling injury through biotechnology. Curr Opin Biotechnol. 2022;78:102790 36116331 10.1016/j.copbio.2022.102790

[3] Franzoni G, Spadafora ND, Sirangelo TM. et al. Biochemical and molecular changes in peach fruit exposed to cold stress conditions. Mol Hortic. 2023;3:24 37953307 10.1186/s43897-023-00073-0 PMC10641970

[4] Sati H, Oberoi HS, Pareek S. Is ATP a signaling regulator for postharvest chilling tolerance in fruits? Hortic Res. 2024;11:uhae204 39286356 10.1093/hr/uhae204 PMC11404123

[5] Min DD, Li FJ, Ali M. et al. Application of methyl jasmonate to control chilling tolerance of postharvest fruit and vegetables: a meta-analysis and eliciting metabolism review. Crit Rev Food Sci Nutr. 2023;64:12878–91 37702765 10.1080/10408398.2023.2258201

[6] Song CB, Zhou C, Pan YY. et al. A new regulatory network controls chilling injury in peach fruit by γ-aminobutyric acid. Food Secur. 2023;12:696 10.3390/foods12040696 PMC9955076 36832770

[7] Ai SJ, Xu SM, Wu CX. et al. Novel insights into modified atmosphere mediated cold tolerance in peach fruit during postharvest storage. Postharvest Biol Technol. 2024;218:113187

[8] Wang LH, Chen H, Chen GY. et al. Transcription factor SlWRKY50 enhances cold tolerance in tomato by activating the jasmonic acid signaling. Plant Physiol. 2024a;194:1075–90 37935624 10.1093/plphys/kiad578

[9] Cao SF, Cai YT, Yang ZF. et al. MeJA induces chilling tolerance in loquat fruit by regulating proline and γ-aminobutyric acid contents. Food Chem. 2012;133:1466–70

[10] Li MQ, Zhang XH, Li JQ. et al. GABA primes defense responses against *Botrytis cinerea* in tomato fruit by modulating ethylene and JA signaling pathways. Postharvest Biol Technol. 2024;208:112665

[11] Vaezi S, Asghari M, Farokhzad A. et al. Exogenous methyl jasmonate enhances phytochemicals and delays senescence in harvested strawberries by modulating GABA shunt pathway. Food Chem. 2022;393:133418 35691062 10.1016/j.foodchem.2022.133418

[12] Rodrigues C, Gaspar PD, Simoes MP. et al. Review on techniques and treatments toward the mitigation of the chilling injury of peaches. J Food Process Preserv. 2022;46:12

[13] Liu GS, Fu DQ, Zhong CF. et al. Transcriptome combined with long non-coding RNA analysis reveals the potential molecular mechanism of high-CO_2_ treatment in delaying postharvest strawberry fruit ripening and senescence. Sci Hortic. 2024a;323:9

[14] Romero I, Alegria-Carrasco E, de Pradena AG. et al. WRKY transcription factors in the response of table grapes (cv. Autumn Royal) to high CO_2_ levels and low temperature. Postharvest Biol Technol. 2019;150:42–51

[15] Vazquez-Hernandez M, Blanch M, Sanchez-Ballesta MT. et al. High CO_2_ alleviates cell ultrastructure damage in Autumn Royal table grapes by modulating fatty acid composition and membrane and cell oxidative status during long-term cold storage. Postharvest Biol Technol. 2020;160:11

[16] Pan CZ, Zhang H, Ma QM. et al. Role of ethylene biosynthesis and signaling in elevated CO_2_-induced heat stress response in tomato. Planta. 2019;250:563–72 31123806 10.1007/s00425-019-03192-5

[17] Zou XL, Shen QJX, Neuman D. An ABA inducible WRKY gene integrates responses of creosote bush (*Larrea tridentata*) to elevated CO_2_ and abiotic stresses. Plant Sci. 2007;172:997–1004

[18] Hu ZJ, Ma QM, Foyer CH. et al. High CO_2_- and pathogen-driven expression of the carbonic anhydrase βCA3 confers basal immunity in tomato. New Phytol. 2021a;229:2827–43 33206385 10.1111/nph.17087

[19] Wu W, Wang MM, Gong H. et al. High CO_2_/hypoxia-induced softening of persimmon fruit is modulated by DkERF8/16 and DkNAC9 complexes. J Exp Bot. 2020;71:2690–700 31926021 10.1093/jxb/eraa009 PMC7210769

[20] Zhu QG, Gong ZY, Huang JW. et al. High-CO_2_/hypoxia-responsive transcription factors DkERF24 and DkWRKY1 interact and activate *DkPDC2* promoter. Plant Physiol. 2019;180:621–33 30850469 10.1104/pp.18.01552 PMC6501092

[21] Zhu QG, Gong ZY, Wang MM. et al. A transcription factor network responsive to high CO_2_/hypoxia is involved in deastringency in persimmon fruit. J Exp Bot. 2018;69:2061–70 29390151 10.1093/jxb/ery028 PMC6018754

[22] Romero I, Vazquez-Hernandez M, Escribano MI. et al. Expression profiles and DNA-binding affinity of five ERF genes in bunches of *Vitis vinifera* cv. Cardinal treated with high levels of CO_2_ at low temperature. Front Plant Sci. 2016;7:1748 27965678 10.3389/fpls.2016.01748 PMC5124697

[23] Zhao ML, Wang JN, Shan W. et al. Induction of jasmonate signalling regulators MaMYC2s and their physical interactions with MaICE1 in methyl jasmonate-induced chilling tolerance in banana fruit. Plant Cell Environ. 2013;36:30–51 22651394 10.1111/j.1365-3040.2012.02551.x

[24] Ming RH, Zhang Y, Wang Y. et al. The JA-responsive *MYC2-BADH-like* transcriptional regulatory module in *Poncirus trifoliata* contributes to cold tolerance by modulation of glycine betaine biosynthesis. New Phytol. 2021;229:2730–50 33131086 10.1111/nph.17063

[25] Hu YF, Zhang HJ, Gu B. et al. The transcription factor VaMYC2 from Chinese wild *Vitis amurensis* enhances cold tolerance of grape (*V. vinifera*) by up-regulating *VaCBF1* and *VaP5CS*. Plant Physiol Biochem. 2022;192:218–29 36272189 10.1016/j.plaphy.2022.10.011

[26] Wang K, Yin XR, Zhang B. et al. Transcriptomic and metabolic analyses provide new insights into chilling injury in peach fruit. Plant Cell Environ. 2017;40:1531–51 28337785 10.1111/pce.12951

[27] Zheng YQ, Liu ZN, Wang H. et al. Transcriptome and genome analysis to identify C2H2 genes participating in low-temperature conditioning-alleviated postharvest chilling injury of peach fruit. Food Qual Saf. 2022;6:1–10

[28] Toreti A, Deryng D, Tubiello FN. et al. Narrowing uncertainties in the effects of elevated CO_2_ on crops. Nat Food. 2020;1:775–82 37128059 10.1038/s43016-020-00195-4

[29] Wang J, Luo Q, Liang X. et al. Glucose-G protein signaling plays a crucial role in tomato resilience to high temperature and elevated CO_2_. Plant Physiol. 2024b;195:1025–37 38447060 10.1093/plphys/kiae136

[30] Abdelhakim LOA, Zhou R, Ottosen CO. Physiological responses of plants to combined drought and heat under elevated CO_2_. Agronomy-Basel. 2022;12:2526

[31] Foyer CH, Noctor G. Redox homeostasis and signaling in a higher-CO_2_ world. Annu Rev Plant Biol. 2020;71:157–82 32442392 10.1146/annurev-arplant-050718-095955

[32] Zhou R, Yu XQ, Wen JQ. et al. Interactive effects of elevated CO_2_ concentration and combined heat and drought stress on tomato photosynthesis. BMC Plant Biol. 2020;20:260 32505202 10.1186/s12870-020-02457-6 PMC7276063

[33] Li X, Ahammed GJ, Li ZX. et al. Decreased biosynthesis of jasmonic acid via lipoxygenase pathway compromised caffeine-induced resistance to *Colletotrichum gloeosporioides* under elevated CO_2_ in tea seedlings. Phytopathology. 2016;106:1270–7 27392179 10.1094/PHYTO-12-15-0336-R

[34] Lu CK, Qi JF, Hettenhausen C. et al. Elevated CO_2_ differentially affects tobacco and rice defense against lepidopteran larvae via the jasmonic acid signaling pathway. J Integr Plant Biol. 2018;60:412–31 29319235 10.1111/jipb.12633

[35] Kazan K . Plant-biotic interactions under elevated CO_2_: a molecular perspective. Environ Exp Bot. 2018;153:249–61

[36] Deewatthanawong R, Rowell P, Watkins CB. γ-Aminobutyric acid (GABA) metabolism in CO_2_ treated tomatoes. Postharvest Biol Technol. 2010;57:97–105

[37] Wang D, Li D, Xu YQ. et al. Elevated CO_2_ alleviates browning development by modulating metabolisms of membrane lipids, proline, and GABA in fresh-cut Asian pear fruit. Sci Hortic. 2021a;281:109932

[38] Li D, Li L, Xiao GN. et al. Effects of elevated CO_2_ on energy metabolism and γ-aminobutyric acid shunt pathway in postharvest strawberry fruit. Food Chem. 2018;265:281–9 29884384 10.1016/j.foodchem.2018.05.106

[39] Liu TF, Li YX, Shi YJ. et al. γ-Aminobutyric acid mediated by MdCBF3-*MdGAD1* mitigates low temperature damage in apple. Int J Biol Macromol. 2024b;279:135331 39236964 10.1016/j.ijbiomac.2024.135331

[40] Wang ZM, Wong DCJ, Wang Y. et al. GRAS-domain transcription factor PAT1 regulates jasmonic acid biosynthesis in grape cold stress response. Plant Physiol. 2021b;186:1660–78 33752238 10.1093/plphys/kiab142 PMC8260143

[41] An JP, Wang XF, Zhang XW. et al. Apple B-box protein BBX37 regulates jasmonic acid mediated cold tolerance through the JAZ-BBX37-ICE1-CBF pathway and undergoes MIEL1-mediated ubiquitination and degradation. New Phytol. 2021;229:2707–29 33119890 10.1111/nph.17050

[42] Zhou JX, Min DD, Li ZL. et al. Effects of chilling acclimation and methyl jasmonate on sugar metabolism in tomato fruits during cold storage. Sci Hortic. 2021;289:110495

[43] Kidokoro S, Shinozaki K, Yamaguchi-Shinozaki K. Transcriptional regulatory network of plant cold-stress responses. Trends Plant Sci. 2022;27:922–35 35210165 10.1016/j.tplants.2022.01.008

[44] Chen HY, Hsieh EJ, Cheng MC. et al. ORA47 (octadecanoid-responsive AP2/ERF-domain transcription factor 47) regulates jasmonic acid and abscisic acid biosynthesis and signaling through binding to a novel *cis*-element. New Phytol. 2016;211:599–613 26974851 10.1111/nph.13914

[45] Hu CY, Wei CY, Ma QM. et al. Ethylene response factors 15 and 16 trigger jasmonate biosynthesis in tomato during herbivore resistance. Plant Physiol. 2021b;185:1182–97 33793934 10.1093/plphys/kiaa089 PMC8133690

[46] Tan XL, Fan ZQ, Shan W. et al. Association of BrERF72 with methyl jasmonate-induced leaf senescence of Chinese flowering cabbage through activating JA biosynthesis-related genes. Hortic Res. 2018;5:22 29736247 10.1038/s41438-018-0028-z PMC5928098

[47] Qi XN, Xiao YY, Fan ZQ. et al. A banana fruit transcriptional repressor MaERF10 interacts with MaJAZ3 to strengthen the repression of JA biosynthetic genes involved in MeJA-mediated cold tolerance. Postharvest Biol Technol. 2016;120:222–31

[48] Wang JR, Zhang Y, Wang JZ. et al. *SlGAD2* is the target of SlTHM27,positively regulates cold tolerance by mediating anthocyanin biosynthesis in tomato. Hortic Res. 2024c;11:uhae096 10.1093/hr/uhae096 PMC11161262 38855415

[49] Zhu JY, Chen HR, Liu L. et al. JA-mediated MYC2/LOX/AOS feedback loop regulates osmotic stress response in tea plant. Hortic Plant J. 2024a;10:931–46

[50] Zhu XP, Wang MM, Huang Z. et al. The OsMYC2-JA feedback loop regulates diurnal flower-opening time via cell wall loosening in rice. Plant J. 2024b;119:2585–98 38972041 10.1111/tpj.16910

[51] Chen YM, Jin GC, Liu MY. et al. Multiomic analyses reveal key sectors of jasmonate-mediated defense responses in rice. Plant Cell. 2024;36:3362–77 38801741 10.1093/plcell/koae159 PMC11371138

[52] Liu YY, Du MM, Deng L. et al. MYC2 regulates the termination of jasmonate signaling via an autoregulatory negative feedback loop. Plant Cell. 2019;31:106–27 30610166 10.1105/tpc.18.00405 PMC6391702

[53] Takahashi Y, Bosmans KC, Hsu PK. et al. Stomatal CO_2_/bicarbonate sensor consists of two interacting protein kinases, Raf-like HT1 and non-kinase-activity requiring MPK12/MPK4. Sci Adv. 2022;8:eabq6161 36475789 10.1126/sciadv.abq6161 PMC9728965

[54] Wang S, Wang T, Li QQ. et al. Phosphorylation of MdERF17 by MdMPK4 promotes apple fruit peel degreening during light/dark transitions. Plant Cell. 2022;34:1980–2000 35166845 10.1093/plcell/koac049 PMC9048921

[55] Zhu YC, Wang K, Wu CX. et al. DNA hypermethylation associated with the development of temperature-dependent postharvest chilling injury in peach fruit. Postharvest Biol Technol. 2021;181:111645

[56] Dhindsa RS, Plumbdhindsa P, Thorpe TA. Leaf senescence - correlated with increased levels of membrane-permeability and lipid-peroxidation, and decreased levels of superoxide-dismutase and catalase. J Exp Bot. 1981;32:93–101

[57] Wang YS, Ding MD, Gu XG. et al. Analysis of interfering substances in the measurement of malondialdehyde content in plant leaves. Am J Biochem Biotechnol. 2013;9:235–42

[58] Huang D, Xue L, Lu YQ. et al. PpBBX32 and PpZAT5 modulate temperature-dependent and tissue-specific anthocyanin accumulation in peach fruit. Hortic Res. 2024;11:uhae212 39385999 10.1093/hr/uhae212 PMC11462610

[59] Liang L, Zhu JZ, Huang D. et al. Molecular mechanisms underlying natural deficient and ultraviolet-induced accumulation of anthocyanin in the peel of 'Jinxiu' peach. Plant Cell Environ. in press. 2024;47:4833–48 39101482 10.1111/pce.15064

[60] Horsch RB, Fry JE, Hoffmann NL. et al. A simple and general method for transferring genes into plants. Science. 1985;227:1229–31 17757866 10.1126/science.227.4691.1229

[61] Hellens RP, Allan AC, Friel EN. et al. Transient expression vectors for functional genomics, quantification of promoter activity and RNA silencing in plants. Plant Methods. 2005;1:13 16359558 10.1186/1746-4811-1-13 PMC1334188

