# Supplementary material for: PpERF17 alleviates peach fruit postharvest chilling injury under elevated CO2 by activating jasmonic acid and γ-aminobutyric acid biosynthesis
Source: Hortic Res. 2025 Jan 15;12(4):uhaf014. doi: 10.1093/hr/uhaf014 (PMC11908827; doi:10.1093/hr/uhaf014)
Supplement: Web_Material_uhaf014 [file web_material_uhaf014.zip › Suppl Figures-HR.docx]

**
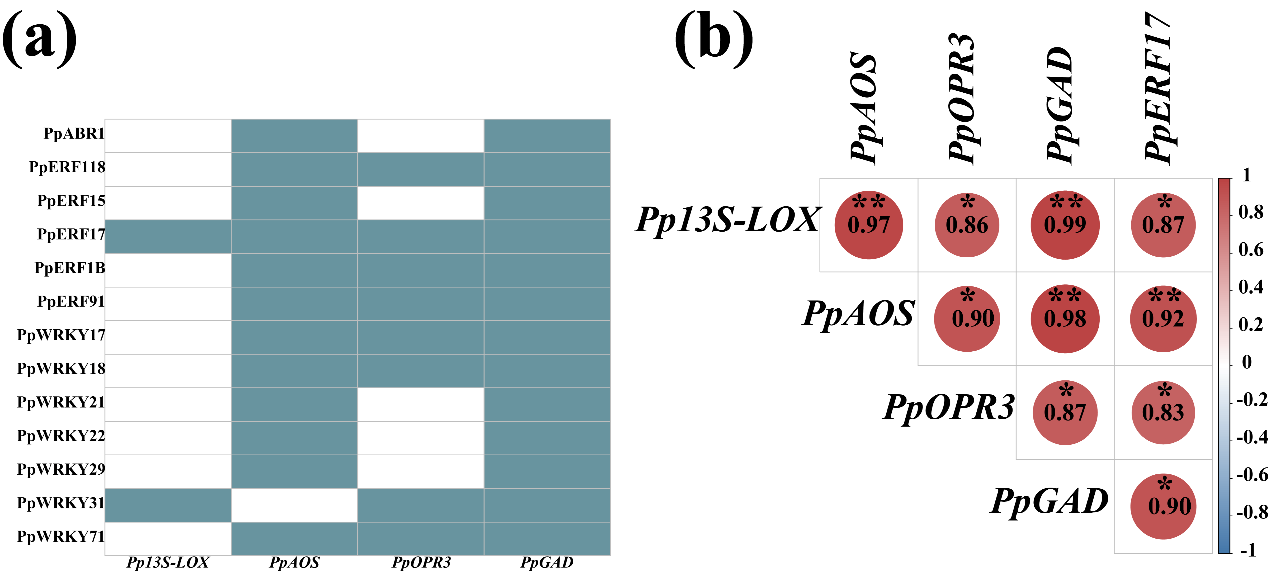
**

**Fig. S1 Identification of candidate transcription factors (TFs) of jasmonic acid (JA) and** **γ-aminobutyric acid (GABA).** (a) TFs targeting biosynthetic pathway of JA and GABA. Blue squares represented the existence of the potential binding sites. (b) Correlation between the expression of *PpERF17* and JA and GABA biosynthetic genes *Pp13S-LOX*, *PpAOS* and *PpOPR3* as well as *PpGAD*. Student’s *t*-test with significance levels indicated as follows: * for *p* < 0.05, ** for *p* < 0.01 and *** for *p* < 0.001. 13S-LOX, 13S-lipoxygenase; AOS, allene oxide synthase; ERF, ethylene response factor; GAD, glutamate decarboxylase; OPR3, 12-oxophytodienoate reductase 3.


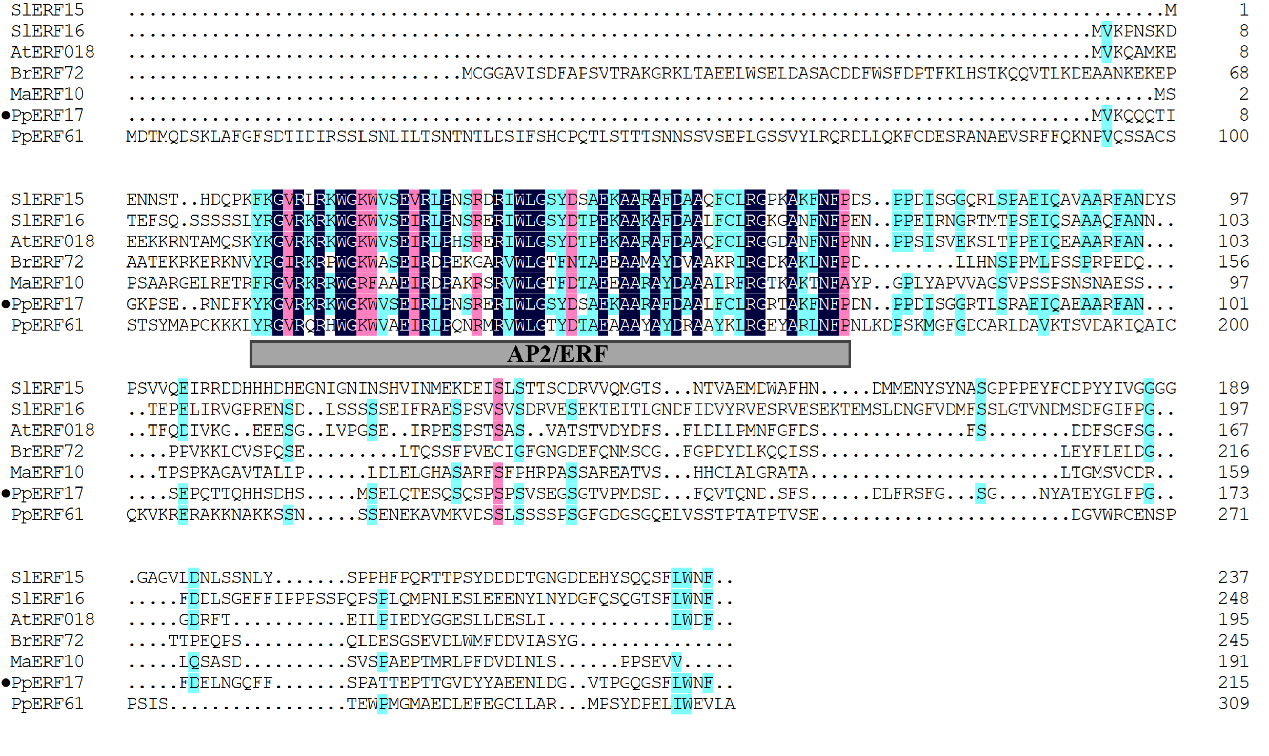


**Fig. S2 Sequence analysis of PpERF17.** Multiple alignment of deduced protein sequence PpERF17 and other plant ERFs reported to be involved in JA synthesis including tomato SlERF15/ERF16, *Arabidopsis* AtERF018, Chinese flowering cabbage BrERF72, banana MaERF10 and peach PpERF61. Alignments were carried out using DNAMAN 6.0. ERF, ethylene response factor; JA, jasmonic acid.


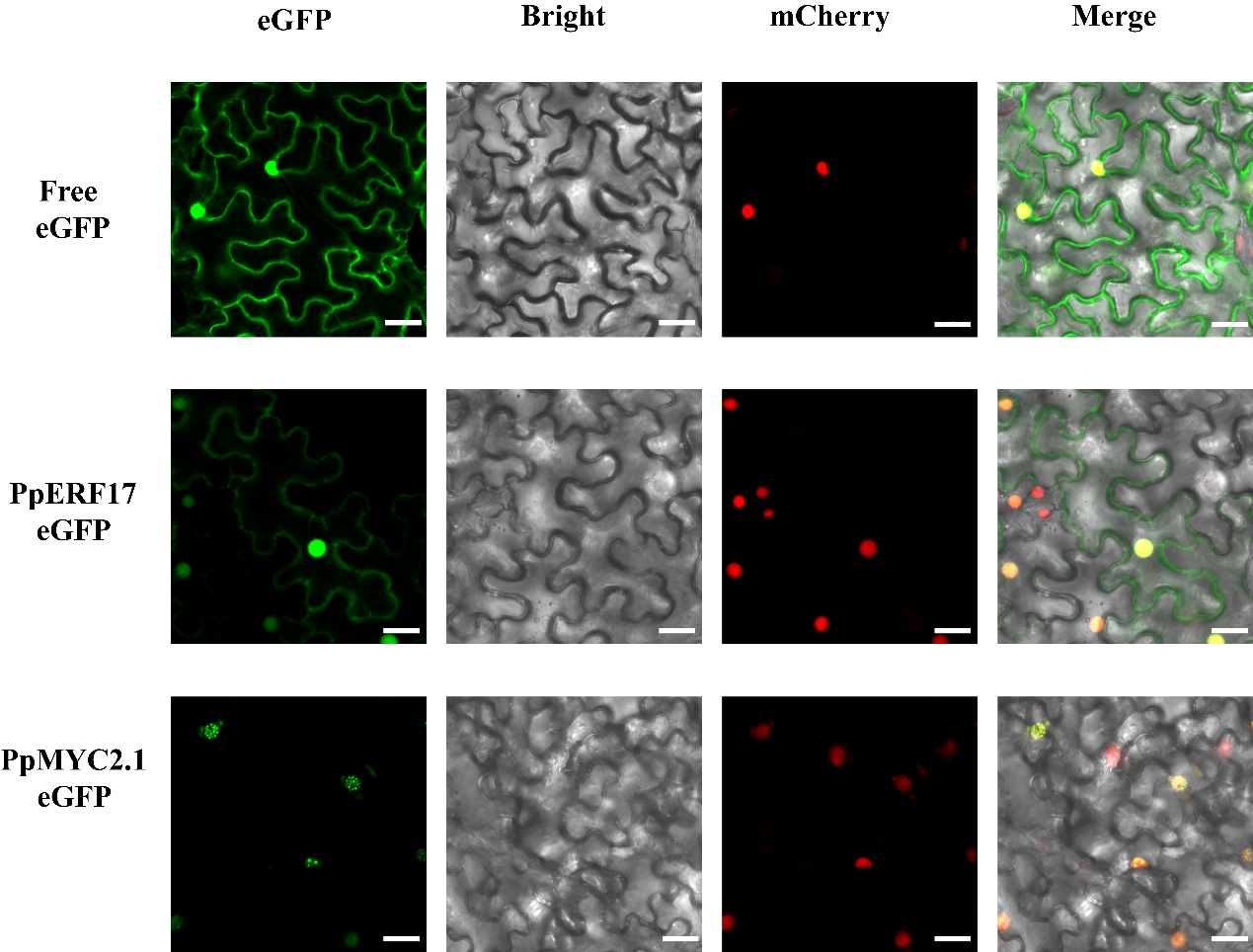


**Fig. S3 Subcellular localization of PpERF17 and PpMYC2.1 in mCherry-NLS transgenic *Nicotiana benthamiana leaves*.** Bar= 20 μm. ERF, ethylene response factor; GFP, Green fluorescent protein; NLS, nuclear localization signal.

**
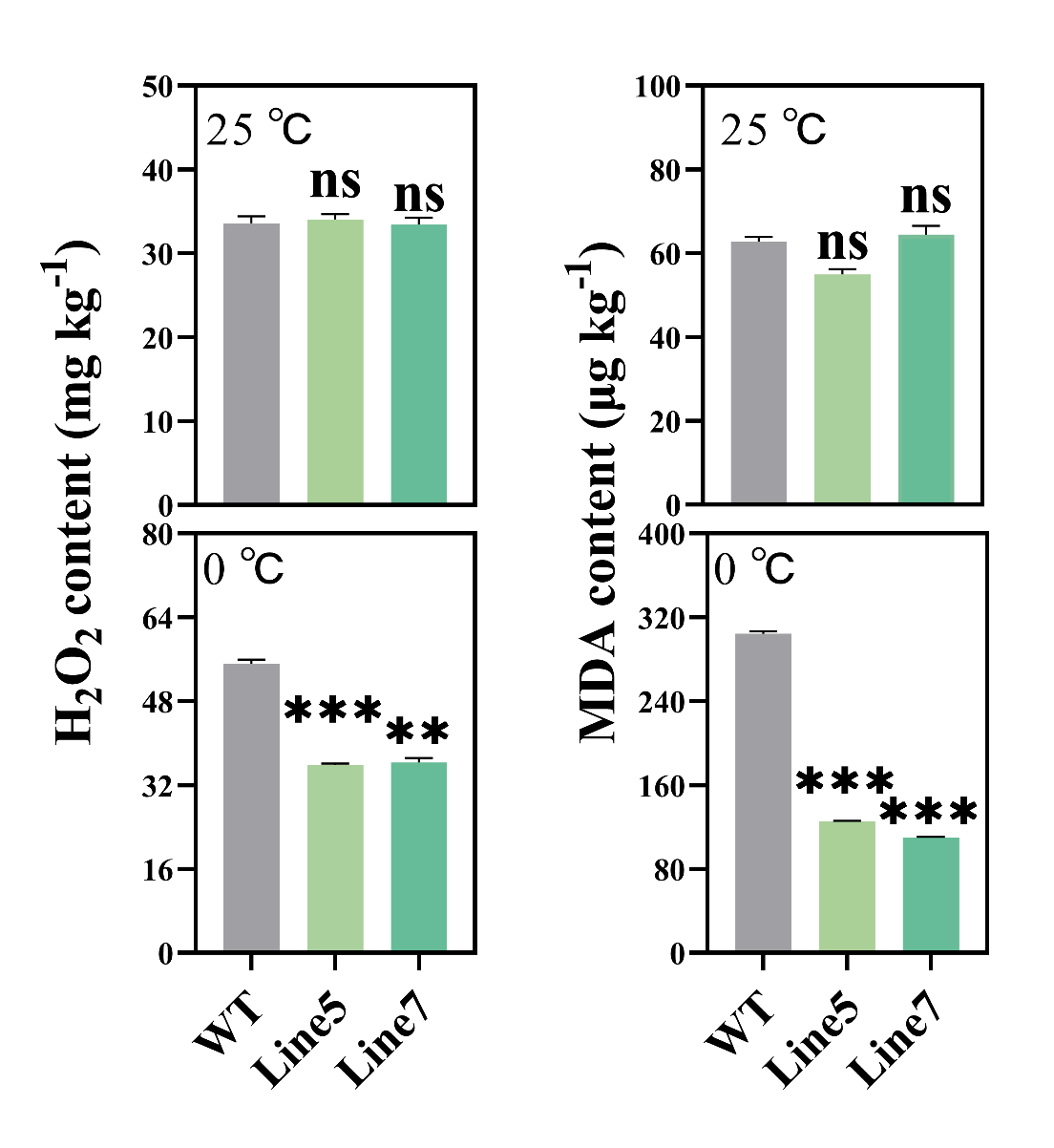
**

**Fig. S4 The contents of hydrogen peroxide (H_2_O_2_) and malondialdehyde (MDA) of transgenic tobacco overexpressing *PpERF17*.** Student’s *t*-test with significance levels indicated as follows: * for *p* < 0.05, ** for *p* < 0.01 and *** for *p* < 0.001. ns, not significant.

**
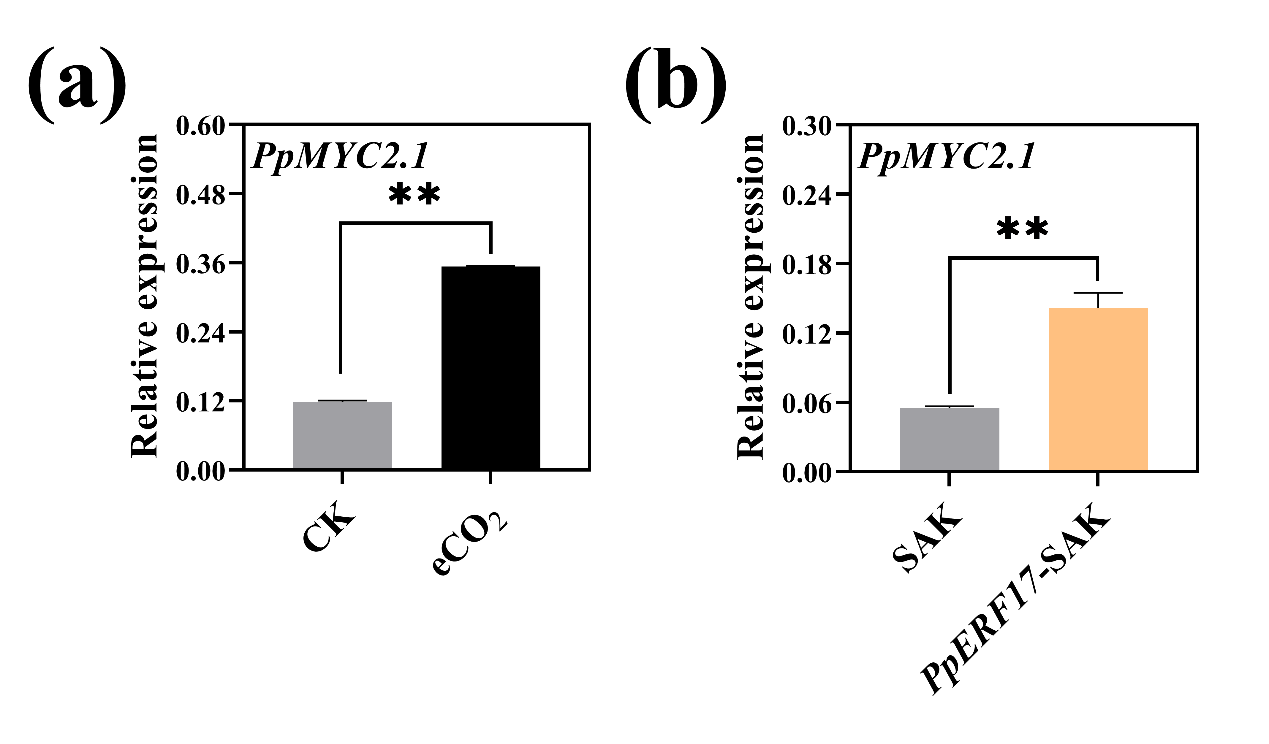
**

**Fig. S5 The expression of *PpMYC2.1* under elevated CO_2_ (eCO_2_) treatment (a) and following transient overexpression of *PpERF17* in peach fruit (b).** Student’s *t*-test with significance levels indicated as follows: * for *p* < 0.05, ** for *p* < 0.01 and *** for *p* < 0.001. CK, 19 % O_2_; eCO_2_, 19 % O_2_ + 10 % CO_2_; ERF, ethylene response factor.

**
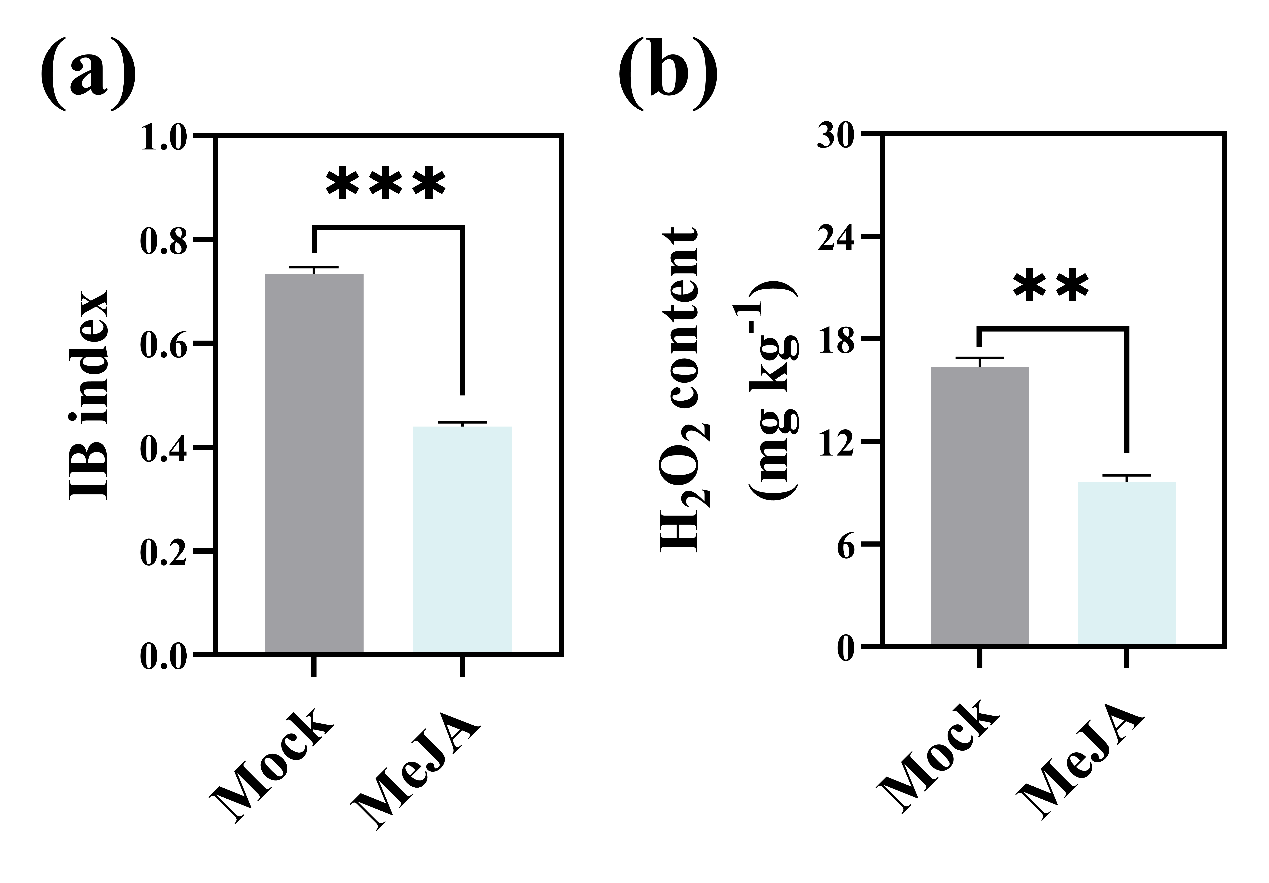
**

**Fig. S6 The internal browning (IB) index (a) and H_2_O_2_ content (b) of ‘Zhonghuashoutao’ (‘ZHST’) peach fruit during 3-day shelf-life following methyl jasmonate (MeJA) treatment.** Student’s *t*-test with significance levels indicated as follows: * for *p* < 0.05, * for *p* < 0.01 and *** for *p* < 0.001.
