# Supplementary material for: PpERF17 alleviates peach fruit postharvest chilling injury under elevated CO2 by activating jasmonic acid and γ-aminobutyric acid biosynthesis
Source: Hortic Res. 2025 Jan 15;12(4):uhaf014. doi: 10.1093/hr/uhaf014 (PMC11908827; doi:10.1093/hr/uhaf014)
Supplement: Web_Material_uhaf014 [file web_material_uhaf014.zip › Suppl Tables-HR.docx]

**Table S1.** **List of transcription factors with potential binding sequences in the promoters of jasmonic acid (JA) and γ-aminobutyric acid (GABA) biosynthetic genes.**

| **TF name** | **family** | **promoter name** | **start** | **stop** | **strand** | **score** | **p-value** | **q-value** | **binding sequence** |
| --- | --- | --- | --- | --- | --- | --- | --- | --- | --- |
| PpABR1 | ERF | PpGAD | 444 | 462 | - | 18.0938 | 0.000000432 | 0.00604 | GGCAATGGTGGCGGCTGGG |
| PpABR1 | ERF | PpGAD | 433 | 451 | - | 17.0781 | 0.00000086 | 0.00604 | CGGCTGGGCGGCGGCGGTG |
| PpABR1 | ERF | PpGAD | 447 | 465 | - | 16.2031 | 0.0000015 | 0.00604 | GTGGGCAATGGTGGCGGCT |
| PpABR1 | ERF | PpGAD | 436 | 454 | - | 16.1562 | 0.00000155 | 0.00604 | TGGCGGCTGGGCGGCGGCG |
| PpABR1 | ERF | PpGAD | 796 | 814 | + | 14.8281 | 0.00000341 | 0.0106 | GCGGGGTGAGGCGGCCGCC |
| PpABR1 | ERF | PpGAD | 808 | 826 | - | 12.6094 | 0.0000112 | 0.0292 | TCGTACTTCGACGGCGGCC |
| PpABR1 | ERF | PpGAD | 430 | 448 | - | 11.1719 | 0.0000225 | 0.05 | CTGGGCGGCGGCGGTGGTA |
| PpABR1 | ERF | PpGAD | 805 | 823 | - | 10.6562 | 0.0000286 | 0.05 | TACTTCGACGGCGGCCGCC |
| PpABR1 | ERF | PpAOS | 1866 | 1884 | - | 10.6406 | 0.0000288 | 0.05 | GAGTGTGAAGACGGTGGGG |
| PpABR1 | ERF | PpAOS | 1045 | 1063 | + | 10.1719 | 0.0000356 | 0.0556 | TTAACAGTATACGGCGGGG |
| PpABR1 | ERF | PpAOS | 141 | 159 | + | 9.07812 | 0.0000573 | 0.0813 | GATAAAGTTGACGGCAGGG |
| PpABR1 | ERF | PpAOS | 453 | 471 | + | 8.59375 | 0.0000702 | 0.0912 | ATAGGCAGTGACGGAGCCA |
| PpERF118 | ERF | PpGAD | 432 | 446 | + | 25.0156 | 8.13E-10 | 1.27E-05 | CCACCGCCGCCGCCC |
| PpERF118 | ERF | PpGAD | 429 | 443 | + | 21.8906 | 2.51E-08 | 0.000196 | CTACCACCGCCGCCG |
| PpERF118 | ERF | PpGAD | 443 | 457 | + | 15.5781 | 0.00000206 | 0.0107 | GCCCAGCCGCCACCA |
| PpERF118 | ERF | PpGAD | 804 | 818 | + | 13.75 | 0.00000553 | 0.0216 | AGGCGGCCGCCGTCG |
| PpERF118 | ERF | PpOPR3 | 1848 | 1862 | + | 12.8594 | 0.00000865 | 0.0229 | CGACCACAGCCGTCG |
| PpERF118 | ERF | PpGAD | 426 | 440 | + | 12.8281 | 0.00000879 | 0.0229 | CAACTACCACCGCCG |
| PpERF118 | ERF | PpGAD | 446 | 460 | + | 12.125 | 0.0000124 | 0.0242 | CAGCCGCCACCATTG |
| PpERF118 | ERF | PpAOS | 943 | 957 | + | 12.125 | 0.0000124 | 0.0242 | CTAACTCCGCCACTA |
| PpERF118 | ERF | PpAOS | 1053 | 1067 | - | 11.1562 | 0.0000194 | 0.0337 | CTTTCCCCGCCGTAT |
| PpERF118 | ERF | PpGAD | 804 | 818 | - | 10.4062 | 0.0000271 | 0.0423 | CGACGGCGGCCGCCT |
| PpERF118 | ERF | PpGAD | 440 | 454 | + | 9.9375 | 0.0000332 | 0.0471 | GCCGCCCAGCCGCCA |

**Table S1** (continued)

| **TF name** | **family** | **promoter name** | **start** | **stop** | **strand** | **score** | **p-value** | **q-value** | **binding sequence** |
| --- | --- | --- | --- | --- | --- | --- | --- | --- | --- |
| PpERF118 | ERF | PpGAD | 801 | 815 | - | 9.125 | 0.0000467 | 0.0608 | CGGCGGCCGCCTCAC |
| PpERF118 | ERF | PpGAD | 901 | 915 | - | 8.53125 | 0.0000595 | 0.0714 | GCACCAGAGCCGCGT |
| PpERF118 | ERF | PpGAD | 801 | 815 | + | 7.46875 | 0.0000901 | 0.0962 | GTGAGGCGGCCGCCG |
| PpERF118 | ERF | PpOPR3 | 1778 | 1792 | + | 7.3125 | 0.0000957 | 0.0962 | CGACCACCACCATTA |
| PpERF118 | ERF | PpOPR3 | 1851 | 1865 | + | 7.23438 | 0.0000986 | 0.0962 | CCACAGCCGTCGGAT |
| PpERF15 | ERF | PpGAD | 432 | 451 | + | 19.7344 | 9.79E-08 | 0.00153 | CCACCGCCGCCGCCCAGCCG |
| PpERF15 | ERF | PpAOS | 1868 | 1887 | + | 15.0938 | 0.00000464 | 0.0362 | CCACCGTCTTCACACTCACC |
| PpERF15 | ERF | PpGAD | 435 | 454 | + | 13.8906 | 0.00000919 | 0.0439 | CCGCCGCCGCCCAGCCGCCA |
| PpERF15 | ERF | PpGAD | 446 | 465 | + | 13.5 | 0.0000112 | 0.0439 | CAGCCGCCACCATTGCCCAC |
| PpERF15 | ERF | PpGAD | 810 | 829 | + | 12.6406 | 0.0000169 | 0.0528 | CCGCCGTCGAAGTACGAAAC |
| PpERF15 | ERF | PpAOS | 138 | 157 | - | 8.64062 | 0.0000748 | 0.165 | CTGCCGTCAACTTTATCTAA |
| PpERF15 | ERF | PpGAD | 1764 | 1783 | + | 8.04688 | 0.0000897 | 0.165 | GCACCGTCCATGTTCCAACT |
| PpERF15 | ERF | PpAOS | 450 | 469 | - | 7.95312 | 0.0000922 | 0.165 | GCTCCGTCACTGCCTATATG |
| PpERF15 | ERF | PpGAD | 438 | 457 | + | 7.85938 | 0.0000948 | 0.165 | CCGCCGCCCAGCCGCCACCA |
| PpERF17 | ERF | PpGAD | 435 | 448 | - | 17.875 | 0.00000036 | 0.00563 | CTGGGCGGCGGCGG |
| PpERF17 | ERF | Pp13S-LOX | 1766 | 1779 | + | 15.3438 | 0.00000391 | 0.0175 | GTGGACGGTGAGAT |
| PpERF17 | ERF | PpGAD | 1761 | 1774 | - | 15.2188 | 0.00000433 | 0.0175 | ATGGACGGTGCCTT |
| PpERF17 | ERF | PpOPR3 | 1692 | 1705 | - | 15.1719 | 0.00000448 | 0.0175 | ATGGCCGGTGTCGT |
| PpERF17 | ERF | PpAOS | 1132 | 1145 | - | 13.8906 | 0.000011 | 0.0301 | TTGGCCGGTAGGGG |
| PpERF17 | ERF | PpGAD | 807 | 820 | - | 13.7031 | 0.0000125 | 0.0301 | TTCGACGGCGGCCG |
| PpERF17 | ERF | PpAOS | 1865 | 1878 | - | 13.5312 | 0.0000139 | 0.0301 | GAAGACGGTGGGGT |
| PpERF17 | ERF | PpAOS | 147 | 160 | + | 13.3594 | 0.0000154 | 0.0301 | GTTGACGGCAGGGG |
| PpERF17 | ERF | PpAOS | 1367 | 1380 | - | 12.8906 | 0.0000203 | 0.0354 | GTGGACGGAGTTTG |

**Table S1** (continued)

| **TF name** | **family** | **promoter name** | **start** | **stop** | **strand** | **score** | **p-value** | **q-value** | **binding sequence** | |
| --- | --- | --- | --- | --- | --- | --- | --- | --- | --- | --- |
| PpERF17 | ERF | PpGAD | 432 | 445 | - | 12.6875 | 0.0000228 | 0.0358 | GGCGGCGGCGGTGG |  |
| PpERF17 | ERF | PpOPR3 | 1771 | 1784 | - | 12.25 | 0.0000291 | 0.0414 | GTGGTCGGCAACTG | |
| PpERF17 | ERF | PpAOS | 1237 | 1250 | + | 11.8438 | 0.000036 | 0.0469 | TTGGGCGGCAGTGA | |
| PpERF17 | ERF | PpOPR3 | 1841 | 1854 | - | 11.5625 | 0.0000415 | 0.05 | GTGGTCGGTTTCTG | |
| PpERF17 | ERF | PpAOS | 1271 | 1284 | + | 11.1719 | 0.0000502 | 0.0561 | GTGGTCGGAAGGGA | |
| PpERF17 | ERF | PpGAD | 429 | 442 | - | 10.9219 | 0.0000564 | 0.0589 | GGCGGCGGTGGTAG | |
| PpERF1B | ERF | PpGAD | 429 | 456 | + | 24.2344 | 2.9E-09 | 4.39E-05 | CTACCACCGCCGCCGCCCAGCCGCCACC | |
| PpERF1B | ERF | PpGAD | 440 | 467 | + | 23.0156 | 1.03E-08 | 7.82E-05 | GCCGCCCAGCCGCCACCATTGCCCACCA | |
| PpERF1B | ERF | PpGAD | 432 | 459 | + | 13.2188 | 0.00000504 | 0.0255 | CCACCGCCGCCGCCCAGCCGCCACCATT | |
| PpERF1B | ERF | PpGAD | 426 | 453 | + | 10.6719 | 0.0000108 | 0.0386 | CAACTACCACCGCCGCCGCCCAGCCGCC | |
| PpERF1B | ERF | PpGAD | 801 | 828 | + | 9.45312 | 0.000015 | 0.0386 | GTGAGGCGGCCGCCGTCGAAGTACGAAA | |
| PpERF1B | ERF | PpGAD | 791 | 818 | - | 8.98438 | 0.0000171 | 0.0386 | CGACGGCGGCCGCCTCACCCCGCTGATG | |
| PpERF1B | ERF | PpGAD | 443 | 470 | + | 8.82812 | 0.0000178 | 0.0386 | GCCCAGCCGCCACCATTGCCCACCACTG | |
| PpERF1B | ERF | PpOPR3 | 1277 | 1304 | + | 7.60938 | 0.0000251 | 0.0475 | ACCCAGCAGCTGCCTGGGCTCTCTTCCT | |
| PpERF1B | ERF | PpAOS | 940 | 967 | + | 6.26562 | 0.0000368 | 0.062 | TTTCTAACTCCGCCACTACATATAGGAA | |
| PpERF1B | ERF | PpAOS | 1381 | 1408 | - | 4.45312 | 0.0000615 | 0.0826 | ACTTTTTAGTCGCCCTCAAGGCCAGACC | |
| PpERF1B | ERF | PpOPR3 | 1848 | 1875 | + | 4.20312 | 0.0000659 | 0.0826 | CGACCACAGCCGTCGGATCGCAGACGGC | |
| PpERF1B | ERF | PpAOS | 1890 | 1917 | - | 4.15625 | 0.0000668 | 0.0826 | GTTTGGTGGCCGCCTTATTTATAGTGGG | |
| PpERF1B | ERF | PpOPR3 | 1354 | 1381 | + | 3.9375 | 0.0000709 | 0.0826 | GGTCTTGCGCTGCCAGTGCTCTTAAACA | |
| PpERF1B | ERF | PpGAD | 888 | 915 | - | 2.76562 | 0.0000969 | 0.105 | GCACCAGAGCCGCGTCCATGACGTAACC | |
| PpERF91 | ERF | PpGAD | 428 | 444 | - | 24.9531 | 2.18E-09 | 3.39E-05 | GCGGCGGCGGTGGTAGT | |
| PpERF91 | ERF | PpGAD | 425 | 441 | - | 16.3906 | 0.00000108 | 0.00663 | GCGGCGGTGGTAGTTGG | |
| PpERF91 | ERF | PpGAD | 439 | 455 | - | 16.0625 | 0.00000128 | 0.00663 | GTGGCGGCTGGGCGGCG | |

**Table S1** (continued)

| **TF name** | **family** | **promoter name** | **start** | **stop** | **strand** | **score** | **p-value** | **q-value** | **binding sequence** |
| --- | --- | --- | --- | --- | --- | --- | --- | --- | --- |
| PpERF91 | ERF | PpGAD | 442 | 458 | - | 14.7656 | 0.00000243 | 0.00891 | ATGGTGGCGGCTGGGCG |
| PpERF91 | ERF | PpGAD | 803 | 819 | - | 14.2812 | 0.00000305 | 0.00891 | TCGACGGCGGCCGCCTC |
| PpERF91 | ERF | PpOPR3 | 1847 | 1863 | - | 13.6719 | 0.00000404 | 0.00891 | CCGACGGCTGTGGTCGG |
| PpERF91 | ERF | PpGAD | 803 | 819 | + | 13.6094 | 0.00000415 | 0.00891 | GAGGCGGCCGCCGTCGA |
| PpERF91 | ERF | PpGAD | 800 | 816 | - | 13.3906 | 0.00000457 | 0.00891 | ACGGCGGCCGCCTCACC |
| PpERF91 | ERF | PpGAD | 431 | 447 | - | 11.2031 | 0.0000114 | 0.0197 | TGGGCGGCGGCGGTGGT |
| PpERF91 | ERF | PpGAD | 976 | 992 | + | 7.85938 | 0.000038 | 0.0592 | CAGACGGCAGTGGCTTC |
| PpERF91 | ERF | PpGAD | 481 | 497 | - | 6.04688 | 0.0000675 | 0.0956 | CAGGTGGCAGTGGTTGG |
| PpERF91 | ERF | PpAOS | 1902 | 1918 | + | 5.45312 | 0.0000807 | 0.105 | AAGGCGGCCACCAAACA |
| PpERF91 | ERF | PpAOS | 939 | 955 | - | 5.125 | 0.0000888 | 0.106 | GTGGCGGAGTTAGAAAT |
| PpWRKY17 | WRKY | PpGAD | 543 | 556 | - | 14.5469 | 0.00000756 | 0.12 | TACTTTGACTTTTT |
| PpWRKY17 | WRKY | PpAOS | 1313 | 1326 | + | 12.2812 | 0.0000264 | 0.209 | GTTTTTGACTTTTG |
| PpWRKY17 | WRKY | PpGAD | 319 | 332 | - | 11.4219 | 0.0000402 | 0.212 | GACTTTGACCTTAT |
| PpWRKY17 | WRKY | PpAOS | 822 | 835 | - | 10.6719 | 0.0000558 | 0.221 | TGCTTTGACTTCAA |
| PpWRKY17 | WRKY | PpOPR3 | 1635 | 1648 | - | 9.875 | 0.0000763 | 0.242 | AGCGTTGACTGGAC |
| PpWRKY18 | WRKY | PpGAD | 544 | 554 | + | 12.5938 | 0.0000344 | 0.294 | AAAAGTCAAAG |
| PpWRKY18 | WRKY | PpOPR3 | 1636 | 1646 | + | 12.5 | 0.000037 | 0.294 | TCCAGTCAACG |
| PpWRKY18 | WRKY | PpGAD | 320 | 330 | + | 11.5156 | 0.0000777 | 0.328 | TAAGGTCAAAG |
| PpWRKY18 | WRKY | PpAOS | 180 | 190 | + | 11.4531 | 0.0000825 | 0.328 | CACAGTCAAGC |
| PpWRKY21 | WRKY | PpGAD | 544 | 554 | + | 15.3906 | 0.00000542 | 0.0852 | AAAAGTCAAAG |
| PpWRKY21 | WRKY | PpAOS | 1315 | 1325 | - | 11.9531 | 0.0000333 | 0.238 | AAAAGTCAAAA |
| PpWRKY21 | WRKY | PpGAD | 320 | 330 | + | 10.8594 | 0.0000528 | 0.238 | TAAGGTCAAAG |
| PpWRKY21 | WRKY | PpAOS | 823 | 833 | + | 10.5625 | 0.0000605 | 0.238 | TGAAGTCAAAG |

**Table S1** (continued)

| **TF name** | **family** | **promoter name** | **start** | **stop** | **strand** | **score** | **p-value** | **q-value** | **binding sequence** |
| --- | --- | --- | --- | --- | --- | --- | --- | --- | --- |
| PpWRKY22 | WRKY | PpGAD | 544 | 556 | + | 14.0625 | 0.0000106 | 0.169 | AAAAGTCAAAGTA |
| PpWRKY22 | WRKY | PpAOS | 1313 | 1325 | - | 11.7656 | 0.0000366 | 0.24 | AAAAGTCAAAAAC |
| PpWRKY22 | WRKY | PpGAD | 320 | 332 | + | 10.625 | 0.0000599 | 0.24 | TAAGGTCAAAGTC |
| PpWRKY22 | WRKY | PpAOS | 823 | 835 | + | 10.6094 | 0.0000604 | 0.24 | TGAAGTCAAAGCA |
| PpWRKY29 | WRKY | PpGAD | 544 | 554 | + | 15.0781 | 0.00000469 | 0.0744 | AAAAGTCAAAG |
| PpWRKY29 | WRKY | PpAOS | 1315 | 1325 | - | 12.2031 | 0.0000413 | 0.236 | AAAAGTCAAAA |
| PpWRKY29 | WRKY | PpGAD | 320 | 330 | + | 11.6562 | 0.000054 | 0.236 | TAAGGTCAAAG |
| PpWRKY29 | WRKY | PpAOS | 823 | 833 | + | 11.5156 | 0.0000594 | 0.236 | TGAAGTCAAAG |
| PpWRKY31 | WRKY | PpOPR3 | 1628 | 1646 | - | 13.5507 | 0.000016 | 0.184 | CGTTGACTGGACTCGGGTG |
| PpWRKY31 | WRKY | PpGAD | 312 | 330 | - | 12.2029 | 0.0000348 | 0.184 | CTTTGACCTTATTTTAATC |
| PpWRKY31 | WRKY | PpGAD | 536 | 554 | - | 12.2029 | 0.0000348 | 0.184 | CTTTGACTTTTTTTTTATT |
| PpWRKY31 | WRKY | Pp13S-LOX | 1629 | 1647 | + | 11.5797 | 0.0000477 | 0.189 | CTTTGACTCGGGACCTCCC |
| PpWRKY71 | WRKY | PpGAD | 544 | 556 | - | 14.6719 | 0.00000529 | 0.0713 | TACTTTGACTTTT |
| PpWRKY71 | WRKY | PpAOS | 1313 | 1325 | + | 14.0312 | 0.000009 | 0.0713 | GTTTTTGACTTTT |
| PpWRKY71 | WRKY | PpGAD | 320 | 332 | - | 12.4844 | 0.000035 | 0.185 | GACTTTGACCTTA |
| PpWRKY71 | WRKY | PpAOS | 823 | 835 | - | 11.7969 | 0.0000567 | 0.224 | TGCTTTGACTTCA |
| PpWRKY71 | WRKY | PpOPR3 | 1636 | 1648 | - | 11.2031 | 0.0000824 | 0.261 | AGCGTTGACTGGA |

**Table S2. Primers used for reverse-transcription quantitative PCR (RT-qPCR).**

| **Gene** | **Forward primer (5' to 3′)** | **Reverse primer (5′ to 3′)** |
| --- | --- | --- |
| *Pp13S-LOX* | GACTGTGCAACAGGCATTGG | GCTTAAGAGCTCCGGTTGGT |
| *PpAOS* | GGTTTGTCGGTGAGGATGGT | CAACGGCGAAGACCCTACTT |
| *PpOPR3* | GCAGTAGTTGGAGCCGTAGG | CGGGGCTGAGTAACATGGAG |
| *PpGAD* | AGCCAATGTCCAGGTGTGCT | AGCCAAGGATAGCAGCGAC |
| *PpERF17* | AATCTGGCTTGGCTCCTACG | GGTTGTCTGAGGCTCCGAAT |
| *PpMYC2.1* | AAGTGGGTTCGTGGCCTATG | TAACGGGAGCCATGTTCACC |
| *PpTEF2* | GGTGTGACGATGAAGAGTGATG | TGAAGGAGAGGGAAGGTGAAAG |
| *Nt13S-LOX* | GGCACTCAAGAATCCGCTCT | TAAGCTCAATGGCAACGGGT |
| *NtAOS* | TACGGCAGAGCAAAACGTGA | CGACGGGCTTTCATTTTCCG |
| *NtOPR3* | GCCTAGGCTTAGCGGTTGTT | ATGAACGTCCCCTGATACGC |
| *NtGAD* | GCACCACTTGGAGATGGAGA | CCGGTGACGATATTGGGCTT |
| *NtEF1α* | TGGTTGTGACTTTTGGTCCCA | ACAAACCCACGCTTGAGATCC |

**Table S3. List of primer sequences used for vector construction.**

| **Primer** | **Sequence (5′ - 3′)** |
| --- | --- |
| **SK:** |  |
| PpERF17-SK-FP | aggacagcccaagctgagctcATGGTGAAGCAGCAGCAGACC |
| PpERF17-SK-RP | gataagcttgatatcgaattcTTAGAAATTCCAGAGGAAAGACCC |
| PpMYC2.1-SK-FP | aggacagcccaagctgagctcATGACGGACTATCGGATACCGC |
| PpMYC2.1-SK-RP | gataagcttgatatcgaattcTTACCGGGAGTCGCCGAT |
| **LUC:** |  |
| Pp13S-LOXPro-LUC-FP | ggcgaattgggtaccgggcccCGGAGACTATGACACTAGGTAGTGCC |
| Pp13S-LOXPro-LUC-RP | cgctctagaactagtggatccCTTTTCTGCCTTAATTCTCTACACAAA |
| PpAOSPro-LUC-FP | ggcgaattgggtaccgggcccCTAAAATGGCTCTAAGGAAGGCAGGAG |
| PpAOSPro-LUC-RP | cgctctagaactagtggatccTGGGTATCAAAAAAAGTTGTTATTGTT |
| PpOPR3Pro-LUC-FP | ggcgaattgggtaccgggcccGCCACAAATAATCAAACCAGTC |
| PpOPR3Pro-LUC-RP | cgctctagaactagtggatccTTTTAGTTCAGACACCGATCTCAGATC |
| PpGADPro-LUC-FP | ggcgaattgggtaccgggcccTTACACACAATGCCACATGTCCA |
| PpGADPro-LUC-RP | cgctctagaactagtggatccGGTGATGGATTGATGATGGTGTAGAA |
| PpERF17Pro-LUC-FP | ggcgaattgggtaccgggcccCGTTGATCATGCACGAATTATATTT |
| PpERF17Pro-LUC-RP | cgctctagaactagtggatccGGACGACGAAGAAGACGACTCC |
| **pAbAi:** |  |
| Pp13S-LOXPro-pAbAi-FP | gaaaagcttgaattcgagctcCGGAGACTATGACACTAGGTAGTGCC |
| Pp13S-LOXPro-pAbAi-RP | atacagagcacatgcctcgagCTTTTCTGCCTTAATTCTCTACACAAA |
| PpAOSPro-pAbAi-FP | gaaaagcttgaattcgagctcCTAAAATGGCTCTAAGGAAGGCAGGAG |
| PpAOSPro-pAbAi-RP | atacagagcacatgcctcgagTGGGTATCAAAAAAAGTTGTTATTGTT |
| PpOPR3Pro-pAbAi-FP | gaaaagcttgaattcgagctcGCCACAAATAATCAAACCAGTC |
| PpOPR3Pro-pAbAi-RP | atacagagcacatgcctcgagTTTTAGTTCAGACACCGATCTCAGATC |

**Table S3** (continued)

| **Primer** | **Sequence (5′ - 3′)** |
| --- | --- |
| PpGADPro-pAbAi-FP | gaaaagcttgaattcgagctcTTACACACAATGCCACATGTCCA |
| PpGADPro-pAbAi-RP | atacagagcacatgcctcgagGGTGATGGATTGATGATGGTGTAGAA |
| PpERF17Pro-pAbAi-FP | gaaaagcttgaattcgagctcCGTTGATCATGCACGAATTATATTT |
| PpERF17Pro-pAbAi-RP | atacagagcacatgcctcgagGGACGACGAAGAAGACGACTCC |
| **pGADT7:** |  |
| PpERF17-AD-FP | gccatggaggccagtgaattcATGGTGAAGCAGCAGCAGACC |
| PpERF17-AD-RP | cagctcgagctcgatggatccTTAGAAATTCCAGAGGAAAGACCC |
| PpMYC2.1-AD-FP | gccatggaggccagtgaattcATGACGGACTATCGGATACCGC |
| PpMYC2.1-AD-RP | cagctcgagctcgatggatccTTACCGGGAGTCGCCGAT |
| **eGFP:** |  |
| PpERF17-eGFP-FP | acgggggacgagctcATGGTGAAGCAGCAGCAGACC |
| PpERF17-eGFP-RP | gctcaccatgtcgacGAAATTCCAGAGGAAAGACCC |
| PpMYC2.1-eGFP-FP | acgggggacgagctcATGACGGACTATCGGATACCGC |
| PpMYC2.1-eGFP-RP | gctcaccatgtcgacCCGGGAGTCGCCGAT |
| **pGEX:** |  |
| PpERF17-pGEX-FP | ggatctggttccgcgtggatccATGGTGAAGCAGCAGCAGACC |
| PpERF17-pGEX-RP | cgatgcggccgctcgagtcgacTTAGAAATTCCAGAGGAAAGACCC |
| PpMYC2.1-pGEX-FP | ggatctggttccgcgtggatccATGACGGACTATCGGATACCGC |
| PpMYC2.1-pGEX-RP | cgatgcggccgctcgagtcgacTTACCGGGAGTCGCCGAT |
| **pSAK277:** |  |
| PpERF17-pSAK-FP | actagtggatccaaagaattcATGGTGAAGCAGCAGCAGACC |
| PpERF17-pSAK-RP | gactctagaagtactctcgagTTAGAAATTCCAGAGGAAAGACCC |

**Table S4. List of probe sequences used for electrophoretic mobility shift assay (EMSA).**

| **Probe** | **Sequence (5′ - 3′)** |
| --- | --- |
| **EMSA:** |  |
| PpERF17-Pp13S-LOXPro-biotin-FP | TCTTAAAAACGTGTGGACGGTGAGATCAACAGTACCC |
| PpERF17-Pp13S-LOXPro-biotin-RP | GGGTACTGTTGATCTCACCGTCCACACGTTTTTAAGA |
| PpERF17-Pp13S-LOXPro-mut-FP | TCTTAAAAACGTGAAAAAAAAAAGATCAACAGTACCC |
| PpERF17-Pp13S-LOXPro-mut-RP | GGGTACTGTTGATCTTTTTTTTTTCACGTTTTTAAGA |
| PpERF17-PpAOSPro-biotin-FP | ATGGTTCATGTACCCCACCGTCTTCACACTCACCTTC |
| PpERF17-PpAOSPro-biotin-RP | GAAGGTGAGTGTGAAGACGGTGGGGTACATGAACCAT |
| PpERF17-PpAOSPro-mut-FP | ATGGTTCATGTACCCTTTTTTTTTCACACTCACCTTC |
| PpERF17-PpAOSPro-mut-RP | GAAGGTGAGTGTGAAAAAAAAAGGGTACATGAACCAT |
| PpERF17-PpOPR3Pro-biotin-FP | TAGAAACCAAAACGACACCGGCCATAAATAAATCCGC |
| PpERF17-PpOPR3Pro-biotin-RP | GCGGATTTATTTATGGCCGGTGTCGTTTTGGTTTCTA |
| PpERF17-PpOPR3Pro-mut-FP | TAGAAACCAAAACGATTTTTTTTTTAAATAAATCCGC |
| PpERF17-PpOPR3Pro-mut-RP | GCGGATTTATTTAAAAAAAAAATCGTTTTGGTTTCTA |
| PpERF17-PpGADPro-biotin-FP | CATAAGGTCGGAAGGCACCGTCCATGTTCCAACTTGG |
| PpERF17-PpGADPro-biotin-RP | CCAAGTTGGAACATGGACGGTGCCTTCCGACCTTATG |
| PpERF17-PpGADPro-mut-FP | CATAAAAAAAAAAGGTTTTTTTTTGTTCCAACTTGG |
| PpERF17-PpGADPro-mut-RP | CCAAGTTGGAACAAAAAAAAAACCTTTTTTTTTATG |
| PpMYC2.1-Pp13S-LOXPro-biotin-FP | GAGAAGACAAGGGGACACATGTCCTTTGAACCCAAC |
| PpMYC2.1-Pp13S-LOXPro-biotin-RP | GTTGGGTTCAAAGGACATGTGTCCCCTTGTCTTCTC |
| PpMYC2.1-Pp13S-LOXPro-mut-FP | GAGAAGACAAGGGGAAAAAATCCTTTGAACCCAAC |
| PpMYC2.1-Pp13S-LOXPro-mut-RP | GTTGGGTTCAAAGGATTTTTTCCCCTTGTCTTCTC |

**Table S4** (continued)

| **Probe** | **Sequence (5′ - 3′)** |
| --- | --- |
| PpMYC2.1-PpAOSPro-biotin-FP | CAAAGAGTTATTAGGCACGTTGTGCCAAAACATTTA |
| PpMYC2.1-PpAOSPro-biotin-RP | TAAATGTTTTGGCACAACGTGCCTAATAACTCTTTG |
| PpMYC2.1-PpAOSPro-mut-FP | CAAAGAGTTATTAGGAAAAAGTGCCAAAACATTTA |
| PpMYC2.1-PpAOSPro-mut-RP | TAAATGTTTTGGCACTTTTTCCTAATAACTCTTTG |
| PpMYC2.1-PpOPR3Pro-biotin-FP | GAGAATCTCGATTGCCACGTGTCTTATTCCAGATAA |
| PpMYC2.1-PpOPR3Pro-biotin-RP | TTATCTGGAATAAGACACGTGGCAATCGAGATTCTC |
| PpMYC2.1-PpOPR3Pro-mut-FP | GAGAATCTCGATTGCAAAAATCTTATTCCAGATAA |
| PpMYC2.1-PpOPR3Pro-mut-RP | TTATCTGGAATAAGATTTTTGCAATCGAGATTCTC |
| PpMYC2.1-PpGADPro-biotin-FP | AATTACACACAATGCCACATGTCCACCTCCGGTAAT |
| PpMYC2.1-PpGADPro-biotin-RP | ATTACCGGAGGTGGACATGTGGCATTGTGTGTAATT |
| PpMYC2.1-PpGADPro-mut-FP | AATTACACACAATGCAAAAATCCACCTCCGGTAAT |
| PpMYC2.1-PpGADPro-mut-RP | ATTACCGGAGGTGGATTTTTGCATTGTGTGTAATT |
| PpMYC2.1-PpERF17Pro-biotin-FP | GAGGAATGAACAACACACGTGCTTGAATCACATATT |
| PpMYC2.1-PpERF17Pro-biotin-RP | AATATGTGATTCAAGCACGTGTGTTGTTCATTCCTC |
| PpMYC2.1-PpERF17Pro-mut-FP | GAGGAATGAACAACAAAAAACTTGAATCACATATT |
| PpMYC2.1-PpERF17Pro-mut-RP | AATATGTGATTCAAGTTTTTTGTTGTTCATTCCTC |
